# Elucidation of morphological and physiological traits contributing to high biomass productivity and consistently high yield in the high-yielding rice variety Kitagenki

**DOI:** 10.3389/fpls.2025.1710830

**Published:** 2025-11-19

**Authors:** Atsushi Yagioka, Satoshi Hayashi, Kenji Kimiwada, Motohiko Kondo

**Affiliations:** 1 Hokkaido Agricultural Research Center, National Agriculture and Food Research Organization (NARO), Sapporo, Hokkaido, Japan; 2 College of Agriculture, Food and Environment Sciences, Rakuno Gakuen University, Ebetsu, Hokkaido, Japan; 3 Department of Plant Production Sciences, Graduate School of Bioagricultural Sciences, Nagoya University, Nagoya, Aichi, Japan

**Keywords:** biomass productivity, carbon assimilation, canopy architecture, grain-filling ability, light interception, photosynthesis, stable high yield

## Abstract

**Introduction:**

A high-yielding rice variety (HYV), Kitagenki, in the Hokkaido region has a high yield potential owing to its large sink capacity, high source ability, and grain-filling ability. However, the detailed mechanisms underlying high biomass productivity, a major component of source ability, and stable high yields remain elusive. Thus, we aimed to elucidate the canopy morphological and physiological traits that improve the biomass productivity of Kitagenki and how they contribute to a stably high yield.

**Methods:**

We conducted field experiments over 8 years using three rice varieties (Nanatsuboshi: standard-yielding variety, Kita-aoba, and Kitagenki: HYV) with three replicates.

**Results and discussion:**

Kitagenki stably produced higher gross hulled grain yield than Kita-aoba by 4.9–14.9% (8.9% on average) because of higher filled-grain percentage. During 0–20 days after the full-heading stage (DAH), Kitagenki revealed a markedly higher crop growth rate by 29.7% than Kita-aoba because of a higher net assimilation rate while maintaining leaf area index. During these stages, Kitagenki showed a better canopy architecture, characterized by substantially higher leaf inclination angles of the upper two leaves and narrower leaf blades, which facilitated better light interception inside the canopy and higher ^13^C assimilation of the third and whole leaves than in Kita-aoba. At the single-leaf level, Kitagenki showed a higher photosynthetic rate in the third leaf and higher stomatal conductance. Consequently, adequate carbohydrate supply during the early grain-filling stages in Kitagenki enabled faster translocation into the inferior spikelet, resulting in a higher grain-filling ability than that in Kita-aoba. This further contributed to the higher grain yield per cumulative solar radiation during 0–40 DAH in Kitagenki than in Kita-aoba under fluctuating air temperature. These findings indicate that superior canopy architecture, better light interception inside the canopy, and higher carbon assimilation of lower leaves contribute to high biomass productivity during the early grain-filling stage, leading to high grain-filling ability and a stable high yield in Kitagenki compared to Kita-aoba. These results provide key canopy morphological and physiological traits for breeding future HYV that can break the yield ceiling in cold regions.

## 1 Introduction

Increasing rice production is necessary because rice is a staple food that feeds more than half of the world’s population (Khush, 2005). In Japan, in contrast to the reduced demand for rice as a staple food by approximately 100 thousand t annually, rice production for animal feed has increased from 109 thousand t in 2013 to 803 thousand t in 2022 (Ministry of Agriculture, Forestry and Fisheries, 2025). Increasing rice grain yield by breeding high-yielding rice varieties (HYV) is a promising agronomic strategy to meet this increasing demand.

In Japan, several HYVs have been released, and high grain yields have been reported. For instance, indica-type HYVs: Hokuriku193 (Goto et al., 2009) and Takanari (Imbe et al., 2004) produced a high gross hulled grain yield of over 11 t ha^−1^ in the central and southwestern parts of Japan, where the air temperature during the rice cultivation period exceeds 20°C (Okamura et al., 2022; Kobayashi et al., 2014; Nagata et al., 2016). In contrast, japonica-type HYV: Kitagenki (Ikegaya et al., 2017) showed extremely high yield potentials of 12 t ha^−1^ (14.5 t ha^−1^ of rough grain yield) in the Hokkaido region, the northernmost part of Japan, where the air temperature during their rice cultivation period is <20°C (Yagioka et al., 2022). This yield potential exceeded those of previous japonica-type HYV in the same region (>10 t ha^−1^; Hayashi et al., 2012; Hayashi, 2015) or large grain japonica-type HYV in the Tohoku region, the north-eastern parts of Japan (>9 t ha^−1^; Fukushima et al., 2011; Mae et al., 2006). Despite extensive research on high-yielding traits and/or plant types of indica-type HYV in the warmer regions of Japan (Katsura et al., 2008; Kobayashi et al., 2014; Nagata et al., 2001; Okamura et al., 2018, 2021, 2022; Saitoh et al., 2002; Takai et al., 2006; Taylaran et al., 2009; Yoshinaga et al., 2013), relatively little information is available on japonica-type HYV in the Hokkaido region (Hayashi et al., 2012; Hayashi, 2015; Yagioka et al., 2021). Anzoua et al. (2010) elucidated the historical alterations in plant type along with yield improvement in the Hokkaido region using older rice varieties released from 1905 to 1988. However, the plant types of recent HYV in this region have not been assessed.

From a global perspective, the Hokkaido region (41°2’–45°3’N latitude) is one of the northernmost limits of rice cultivation (Fujino et al., 2019). Similarly, Heilongjiang province in China (43°26’–53°33’N latitude) is considered the northernmost region of rice cultivation in the world (Wang et al., 2024). The simulated yield potentials (rough grain yield) of northeast China including Heilongjiang province is estimated to be 12.3–13.6 t ha^−1^ (Wang et al., 2018). To narrow the yield gap in Heilongjiang province, Jia et al. (2023) evaluated several crop management practices using single variety (japonica-type variety DN427) and achieved the maximum rough grain yield of approximately 11 t ha^−1^ under 200 kgN ha^−1^ at 45°52’ N latitude. Shahbaz Farooq et al. (2021) also compared the growth and grain yield of four japonica rice cultivars in two regions of Heilongjiang province and reported the maximum rough grain yield of 13.4 t ha^−1^ (adjusted to 15% moisture content) using Longdao-18. However, to date, a rough grain yield exceeding 14 t ha^−1^ has not been achieved in the region. Furthermore, although Shahbaz Farooq et al. (2021) analyzed grain yield response in terms of yield components, grain-filling traits, and dry matter production, canopy morphological and physiological traits have not been fully characterized. Therefore, elucidating the plant types of the japonica-type HYV with high yield potentials of 12 t ha^−1^ (14.5 t ha^−1^ of rough grain yield) in the Hokkaido region can provide novel insights on breeding future HYV that can break the yield ceiling in cold regions worldwide.

Sink capacity (total spikelet number × one-grain weight) and grain-filling ability determine grain yield (Yoshinaga et al., 2013; Yagioka et al., 2021). Sink-source relationships influence grain-filling ability (Yagioka et al., 2021). Source can be expressed as source ability, that is, available carbohydrate, which is calculated as non-structural carbohydrate (NSC) at full-heading stage (FH) plus dry matter production (ΔW) during the grain-filling stages (Morita and Nakano, 2011). In the Hokkaido region, our previous study showed that Kitagenki produced 24.6% higher grain yields than the standard yielding variety (SYV): Nanatsuboshi, because of its larger sink capacity. Moreover, the grain yield of Kitagenki was higher than that of the previous HYV: Kita-aoba, owing to the higher grain-filing ability at a large sink capacity, supported by a higher source ability per spikelet and spikelet fertility (Yagioka et al., 2021). Kitagenki showed higher NSC and ΔW during the early grain-filling stages than Kita-aoba, which can partly explain the high grain-filling ability. However, the mechanism by which canopy morphological and physiological traits (e.g., leaf area index [LAI], canopy architecture, leaf morphological traits, light and leaf N distribution inside the canopy, and/or photosynthetic rate) contribute to high biomass productivity in Kitagenki and to a stable high yield remain elusive.

To bridge these knowledge gaps, this study aimed to elucidate the morphological and physiological traits that contribute to high biomass productivity in Kitagenki, and how they contribute to a stably high yield. We compared two HYV, Kitagenki and Kita-aoba, which have varying grain-filling abilities at a large sink capacity (Yagioka et al., 2021). SYV: Nanatsuboshi, a leading rice variety in the Hokkaido region, was compared as a reference variety with a smaller sink capacity than Kitagenki (Yagioka et al., 2021). Through field experiments, we observed a better canopy structure in Kitagenki, characterized by more erect and narrower upper leaves and shorter culm lengths than Kita-aoba (**Supplementary Figure S1**), which might benefit canopy photosynthesis via better light interception and higher photosynthetic performance in lower leaves. From these observations, we hypothesized that the superior canopy architecture and carbon assimilation of the lower leaves improve biomass productivity in Kitagenki.

## 2 Material and methods

### 2.1 Site description and experimental design

Eight years (2014–2021) of field experiments were conducted in an irrigated paddy field at the Hokkaido Agricultural Research Center, NARO, Sapporo, Hokkaido, Japan (43° 0′ N, 141° 25′ E). A randomized complete block design was used with three replicates. Three rice varieties were used: Nanatsuboshi, Kita-aoba, and Kitagenki. The fertilizer application rate was 105 kg N ha^−1^ of ammonium sulfate, 80 kg P_2_O_5_ ha^−1^ of superphosphate, and 80 kg K_2_O ha^−1^ of potassium chloride, which were applied as the basal fertilizer. The plot size was 15.9–63.6 m^2^. All plots were tilled by chisel plowing, followed by rotary tillage immediately after basal fertilizer application, at a tillage depth of approximately 0–15 cm. Following rotary tillage, puddling was performed several days following submergence.

Rice seeds were sown in nursery pods between April 11 and 22, and mature seedlings (28–40 d) were transplanted into fields between May 18 and 22, depending on the year. The planting density was 20.8–23.1 hills m^−2^ (row spacing of 33 cm and hill spacing of 13.1–14.6 cm).

### 2.2 Measurement of climate conditions

Solar radiation and air temperature were monitored at the meteorological station of the Hokkaido Agricultural Research Center, located approximately 1km away from the experimental fields. Specifically, air temperature was measured with a platinum resistance thermometer (Pt100) installed in a ventilated shield. Solar radiation was measured with a pyranometer (CMP-21F, Kipp & Zonen, The Netherlands).

### 2.3 Measurement of lodging score and culm length

At maturity stage (M), lodging score was measured on a scale from zero (no lodging) to five (complete lodging) (Yagioka et al., 2022). At the middle grain-filling stages, the culm length (length from the ground surface to the panicle base) of the longest culm on five continuous hills were measured by ruler (Yagioka et al., 2022).

### 2.4 Measurement of grain yield, sink capacity, and filled-grain percentage

At M, rice plants in the 3.0 m^2^ plot were harvested. After total spikelet number was measured as described in Yagioka et al. (2021), the rough grain was weight as “rough grain yield”. Thereafter the rough grain was hulled and weighed as “gross hulled grain yield”. The sub-samples of hulled grain were screened to 1.8 mm, thereafter thousand grain weight were calculated using the grain thicker than 1.8 mm. Sink capacity was calculated using the following Equation 1 (Akita, 1989; Yoshinaga et al., 2013):

(1) $$\begin{array}{c} \text{Sink capacity}=\text{total spikelet number}\\ \times \text{thousand grain weight}/1000 \end{array}$$

Using the above sample from 3.0 m^2^ plot, filled-grain percentage was calculated using the following Equation 2 (Yagioka et al., 2022):

(2) $$\begin{array}{c} \text{Spikelet number thicker than }1.8\text{ mm}\\ \times 100/\text{total spikelet number} \end{array}$$

GY/R (Nagata et al., 2016) and filled grain percentages/R were also calculated using the following Equation 3, 4:

(3) $$\begin{array}{c} \text{GY}/\text{R}=\text{gross hulled grain yield}/\\ \text{cumulative solar radiation from }0\text{ to }40\text{ DAH} \end{array}$$

(4) $$\begin{array}{c} \text{Filled}\;\text{grain percentage}/\text{R}\\ =\text{filled}\;\text{grain percentage}/\\ \text{cumulative solar radiation from }0\text{ to }40\text{ DAH} \end{array}$$

### 2.5 Measurement of shoot dry matter, panicle dry matter, LAI, and specific leaf N in rice plant

To determine shoot dry matter (SDM), panicle dry weight, LAI, and specific leaf N in rice plant, eight continuous hills in canopy condition were sampled at FH and 20 and 40 DAH. Among the eight hills, the two representative hills with averaged stem number were separated into seven parts: panicle, culm plus leaf sheath, leaf blade at different positions (first, second, third, and fourth plus the remaining leaves counted from the flag leaf), and dead leaf blade. The LAI of the leaf blades was measured using a leaf area meter (LI-3100 C, Meiwa, Tokyo, Japan). At M, rice plants in the 3.0 m^2^ plot were harvested as described in sub-section 2.4, thereafter separated into two parts (rough grains and rice straw). Additionally, the two representative hills with averaged stem number were also sampled at M and separated into seven parts in the same manner as reported above, and LAI was measured. The seven plant parts from the two hills, whole shoots from the remaining six hills, and two plant parts (grain and rice straw) separated in M were oven-dried at 80°C for >48 h, weighed, and summed as the shoot dry matter (Yagioka et al., 2022).

The N concentrations in the leaf blades were measured using a CN analyzer (Vario MAX CNS, Elementar, Hanau, Germany). LAI (Breda, 2003), crop growth rate (CGR; Zhu et al., 2020; Zhang et al., 2025), net assimilation rate (NAR; Zhu et al., 2020; Zhang et al., 2025), mean LAI, panicle growth rate (Okamura et al., 2018), and specific leaf N were calculated using the following Equation 5–10:

(5) LAI=specific leaf area×leaf blade's dry weight per land area

(6) $${\text{CGR}}_{\left(\text{t}1–\text{t}2\right)}=({\text{SDM}}_{2}–{\text{SDM}}_{1})/({\text{t}}_{2}–{\text{t}}_{1})$$

(7) $$\begin{array}{c} {\text{NAR}}_{\left(\text{t}1–\text{t}2\right)}=([{\text{SDM}}_{2}–{\text{SDM}}_{1}]/[{\text{t}}_{2}–{\text{t}}_{1}])\\ \times ([{\text{lnLAI}}_{2}–{\text{lnLAI}}_{1}]/[{\text{LAI}}_{2}–{\text{LAI}}_{1}]) \end{array}$$

(8) $$\text{Mean}-{\text{LAI}}_{\left(\text{t}1–\text{t}2\right)}={\text{CGR}}_{\left(\text{t}1–\text{t}2\right)}/{\text{NAR}}_{\left(\text{t}1–\text{t}2\right)}$$

(9) $$\begin{array}{c} {\text{Panicle growth rate}}_{\left(\text{t}1–\text{t}2\right)}\\ =({\text{panicle dry weight}}_{2}–{\text{panicle dry weight}}_{1})/({\text{t}}_{2}–{\text{t}}_{1}) \end{array}$$

(10) Specific leaf N=leaf N concentration/(specific leaf area×100)

(t_1_ and t_2_: the first and second sampling time, SDM_1_ and SDM_2_: SDM at the first and second sampling time, LAI_1_ and LAI_2_: LAI at the first and second sampling time; panicle dry weight_1_ and panicle dry weight_2_: panicle dry weight at the first and second sampling time).

### 2.6 Measurement of leaf inclination angle, leaf blade length, and leaf blade width

The leaf inclination angles of the upper two leaves were assessed during the early grain-filling stages (0–20 DAH) over 5 years (2016–2020). Fifteen representative hills with averaged stem number were randomly selected, and one representative stem with average leaf size per hill was chosen. Thereafter, the leaf inclination angles (defined as angle from the horizon to the tangent line at the center of the leaf blades) of the first and second leaf blades from flag leaf were measured using a digital angle meter (Shinwa, Niigata, Japan).

The leaf blade length and width of the upper three leaves were measured during the middle grain-filling stages over 3 years (2016–2018). Ten representative leaves per plot with average leaf size were chosen and leaf blade length and width were measured using a ruler and the values were averaged.

### 2.7 Measurement of relative light interception rate

During the early grain-filling stages, the relative light interception rate inside the canopy was measured using solarimeter films (Y-1W, Taisei, Tokyo, Japan) over 3 years (2016–2018). As this film fades as solar radiation is absorbed (Kawamura et al., 2005), the relationship between the fading rate of the film (F) and the cumulative solar radiation were considered in the preliminary experiment. Totally 130 films were attached to a wooden board at 10 cm intervals and subsequently placed horizontally on the soil surface near the meteorological station of the Hokkaido Agricultural Research Center. During 10-day periods, each 10 film samples were taken at totally 13 times, and the absorbance of the films at the initial (D_0_) and the sampling times after exposure (D) were measured using a spectrophotometer (D-Meter, RYO-470, Taisei, Tokyo, Japan), and F was calculated using the following Equation 11 (Kawamura et al., 2005):

(11) $$\text{F}=100\times \text{D}/{\text{D}}_{0}$$

Hourly mean solar radiation was also monitored by the method written in sub-section 2.2, and cumulative intercepted solar radiation between the initial and sampling times was calculated. Thereafter, the quadratic relationship between F and the cumulative intercepted solar radiation were determined and expressed by the following Equation 12:

(12) $$\begin{array}{c} \text{Cumulative intercepted solar radiation}({\text{MJ m}}^{–2}\\ )=-0.0098\times {\text{F}}^{2}–0.2351\times \text{F}+118.63 \end{array}$$

In the main experiment, ten films per plot were attached to a wooden board at 10 cm intervals and subsequently placed horizontally on the inter-row space inside the canopy at three different heights from the ground (60, 35, and 10 cm) for 7–10 days. Furthermore, 20 films were placed outside the canopy (in an open space without shading) in the same field.

After D_0_ and D were measured by the above method, the cumulative intercepted solar radiation was calculated based on the Equation 12. The relative light interception rate was calculated using the following Equation 13:

(13) $$\begin{array}{c} \text{Relative light interception rate}(\%)\\ =\text{cumulative intercepted solar radiation at }\\ \text{each height inside the canopy}\\ \times 100/\text{cumulative intercepted solar }\\ \text{radiation outside the canopy} \end{array}$$

### 2.8 Measurement of leaf photosynthetic rate and stomatal conductance

The leaf photosynthesis rate and stomatal conductance of the upper three leaves were measured during the early grain-filling stages (0–20 DAH) over 2 years (2017–2018). Approximately five leaves of the main stem or equivalent stem, with average leaf color determined by Soil and Plant Analyzer Development values, were selected for each leaf position per plot. A LI6400 (Meiwa, Tokyo, Japan) was used for the measurement during 9:00–12:00 at a temperature of 25°C inside the chamber, 2000 PAR, CO_2_ concentrations of 400 ppm, and a VPD of approximately <1.5 kPa. The measurement was conducted under adequate sunlight on sunny days. Each measurement was conducted when the photosynthetic rate saturated.

### 2.9 ^13^C tracer experiment

In 2020, ^13^C tracer experiment was conducted to compare varietal differences in the carbon assimilation of leaf blades at various positions and their translocation into different parts of rice plants. ^13^CO_2_ was supplied at two different stages: early (12 DAH) and middle (27 DAH) grain-filling stages. The ^13^CO_2_ was generated by adding 10 ml of 7.3M H_3_PO_4_ to 99 atom% Ba_13_CO_3_ inside a canopy frame (60 cm × 30 cm × 105 cm) equipped with a fan. Rice plants on four continuous hills were enclosed within each frame per plot. ^13^C was fed for an hour with gas circulation, based on Sasaki et al. (2005), who reported that ^13^C was completely assimilated after 1 h. At 1 h and 25 h after initiating the ^13^C feeding, rice plant on one hill was sampled at each stage and cut into leaf blades at different positions (first, second, third, and fourth plus the remaining leaf), dead leaf blade, stem (culm and leaf sheath), spikelet on different parts of the panicle (primary rachis branch [PRB] on upper parts [PRB upper], PRB on lower parts [PRB lower], secondary rachis branch [SRB] on upper parts [SRB upper], and SRB on lower parts [SRB lower]), as well as the remaining parts of the panicles. The rice plants were dried at 80°C for >48 h, and their dry weights were measured. The ^13^C concentrations were measured using an ANCA-SL elemental analyzer coupled to a 20–20 mass spectrometer (Europa Scientific, Crewe, UK), and the C concentration was measured using an NC analyzer (Vario MAX CNS, Elementar, Hanau, Germany). The number of spikelets at each position in the panicle was counted to calculate the ^13^C per spikelet.

### 2.10 Grain-filling of the spikelet on various positions in the panicle

To determine the grain-filling pattern of spikelets, the increase of single grain weight at various positions in the panicles were assessed during the grain-filling stages over 2 years (2016–2017). First, panicles whose spikelets on the top flowered at the FH were tagged. Thereafter, three (2017) to four (2016) panicles per plot were sampled at FH, 10, 20, 30, and 40 DAH, as well as M. The panicles were separated into four different parts: (1) PRB upper, (2) PRB lower, (3) SRB upper, and (4) SRB lower, according to the method described by Yagioka et al. (2021). After counting the number of spikelets, they were oven-dried at 80°C for >48 h, weighed, and the single grain weight was calculated.

To determine the contribution of the grain-filling ability at various positions in the panicles to the final grain yield, the filled spikelet number and filled grain weight at M were evaluated over 5 years (2016–2020). The panicle sample at M separated from the two hills with averaged stem number (the method described in subsection 2.5) was further separated into four different parts (PRB upper, PRB lower, SRB upper, and SRB lower), as mentioned above. The spikelet samples were soaked in tap water with a specific gravity of 1.0 to separate filled and unfilled spikelets (Komatsu et al., 1984). After the number of filled spikelets was counted, they were oven-dried at 80°C for >48 h, weighed, and the filled grain weight was calculated.

### 2.11 Statistical analysis

Statistical analysis was conducted using SPSS version 30 (IBM, New York, USA). After the Shapiro-Wilk test was performed to validate normality for three varieties separately, parameters that have non-normal distribution (p < 0.05) for at least one variety were square root transformed. Thereafter, analysis of variance (ANOVA) for the randomized complete block design was conducted using a general linear model. Replication was considered a random factor, whereas year (Y) and variety (V) were considered fixed factors. All data except for ^13^C were analyzed using an ANOVA model, which included Y, replications within Y, V, and Y × V. Tukey’s multiple comparison analysis was performed for the three varieties if significant effects of V were observed in the initial two-way ANOVA. The data of ^13^C was analyzed using an ANOVA model, which included replications and V.

## 3 Results

### 3.1 Mean solar radiation and air temperature

The mean solar radiation and mean air temperature fluctuated over 8 years (**Supplementary Figure S2**). Across three varieties used, the mean solar radiation ranged from 16.9 to 21.0 MJ m^−2^ during transplantation stage (T)–FH, 13.4–19.8 MJ m^−2^ during 0–40 DAH, 10.0–17.2 MJ m^−2^ during 40 DAH–M, and 15.6–20.1 MJ m^−2^ during whole growth stages (T–M). Mean air temperature ranged from 16.5 to 17.9°C during T–FH, 19.1 to 21.7°C during 0–40 DAH, 15.8 to 18.6°C during 40 DAH–M, and 17.6 to 18.9°C during the whole growth stages (T–M).

### 3.2 Growth durations, lodging score, and culm length

Over the 8 years, the three rice varieties had similar full-heading dates and days from T to FH. The harvest day and grain-filling duration of Kitagenki were approximately 5 days later and longer than Nanatsuboshi and comparable with Kita-aoba, except for 2021, when severe lodging urged early Kita-aoba harvest (**Supplementary Table S1**). Lodging score and culm length were markedly lower in Kitagenki than in Kita-aoba and Nanatsuboshi.

### 3.3 Grain yield, sink capacity, and filled-grain percentage

**Figure 1:**
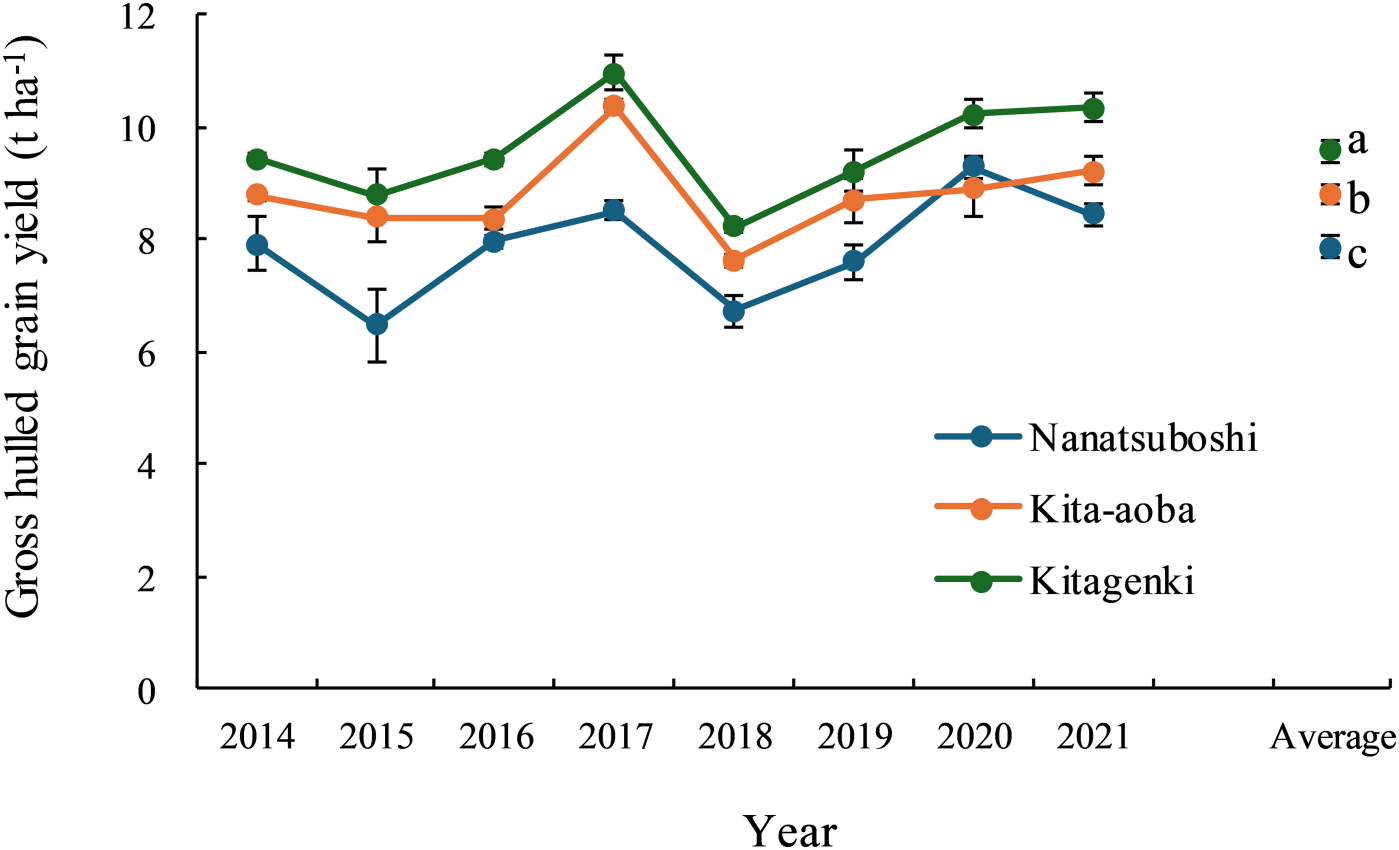
Grain yield variation across 8years (2014–2021). Vertical bars indicate standard errors. Different lowercase letters among the three varieties for the average value across 8 years indicate statistically significant differences (p < 0.05) based on Tukey’s multiple comparison analysis following two-way analysis of variance (ANOVA). The original grain yield data from 2014 to 2018 were obtained from Yagioka et al. (2021) with a modification of the grain moisture content (15% in the current study).

In all years, gross hulled grain yield was consistently higher in Kitagenki than in Kita-aoba by 4.9–14.9% (8.9% on average) and Nanatsuboshi by 10.1–36.0% (21.8% on average) (**Figure 1**). Rough grain yield was also consistently higher in Kitagenki than in Kita-aoba by 3.7–11.9% (6.8% on average) and Nanatsuboshi by 12.6–37.9% (24.0% on average) (**Supplementary Table S1**). Kitagenki produced gross hulled grain yield of >10 t ha^−1^ in three out of 8 years and rough grain yield of >11 t ha^−1^ in six out of 8 years. For an average of 8 years, grain yield was the highest in Kitagenki, followed by Kita-aoba, and was lowest in Nanatsuboshi. The sink capacity of Kitagenki was comparable to that of Kita-aoba but markedly higher than that of Nanatsuboshi over the 8 years (**Supplementary Figure S3A**). The filled-grain percentage was consistently higher in Kitagenki than in Kita-aoba over the 8 years (**Supplementary Figure S3B**).

### 3.4 CGR, mean-LAI, NAR, panicle growth rate, LAI, and specific leaf N

**Figure 2:**
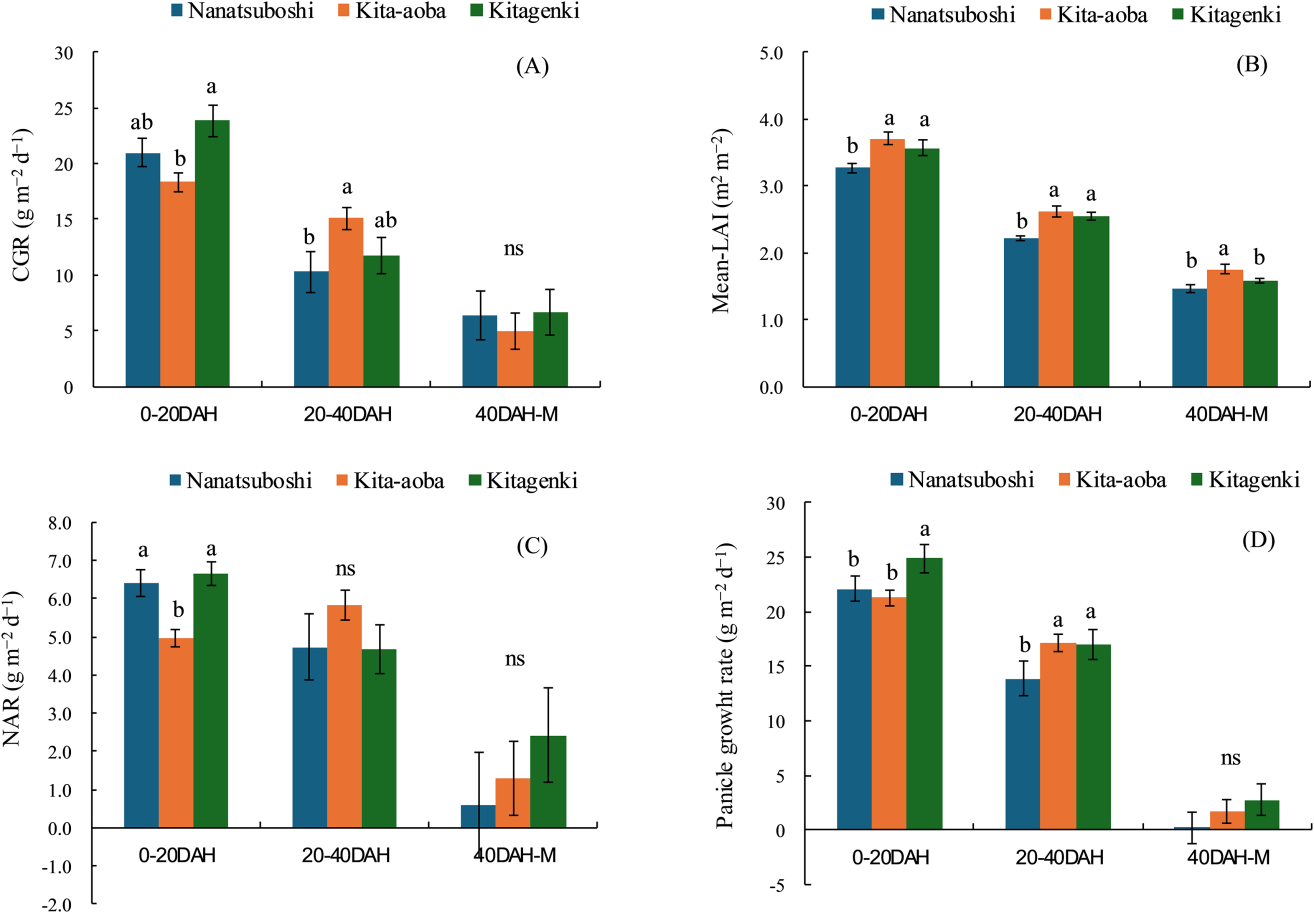
Crop growth rate (CGR) **(A)**, mean leaf area index (Mean-LAI) **(B)**, net assimilation rate (NAR) **(C)**, and panicle growth rate **(D)** during the early (0–20 DAH), middle (20–40 DAH), and late (40 DAH-M) grain-filling stages. The data are averaged over 5 years (2016–2020). Vertical bars indicate the standard error. Different lowercase letters among the three varieties indicate statistically significant differences (p < 0.05) based on Tukey’s multiple comparison analysis following two-way analysis of variance (ANOVA); ns = not significant by two-way ANOVA. DAH, days after full heading stage; M, maturity stage.

Compared with Kita-aoba, Kitagenki exhibited significantly higher CGR, NAR, and panicle growth rates at 0–20 DAH by 29.7%, 33.8%, and 17.2%, respectively, with comparable mean-LAI, averaged across 5 years (**Figure 2**). From 20 to 40 DAH, no substantial differences in the CGR, mean LAI, NAR, or panicle growth rates were noted between Kitagenki and Kita-aoba. Compared to Nanatsuboshi, Kitagenki revealed markedly higher mean LAI and panicle growth rates at 0–20 and 20–40 DAH, respectively. Although there exists yearly variation in CGR and NAR at 0–20 DAH; similar varietal differences were observed in all 5 years, which were stable higher in Kitagenki than in Kita-aoba (**Supplementary Figures S5A, C**). In contrast, varietal difference in mean-LAI fluctuated over 5 years (**Supplementary Figure S5B**).

**Figure 3:**
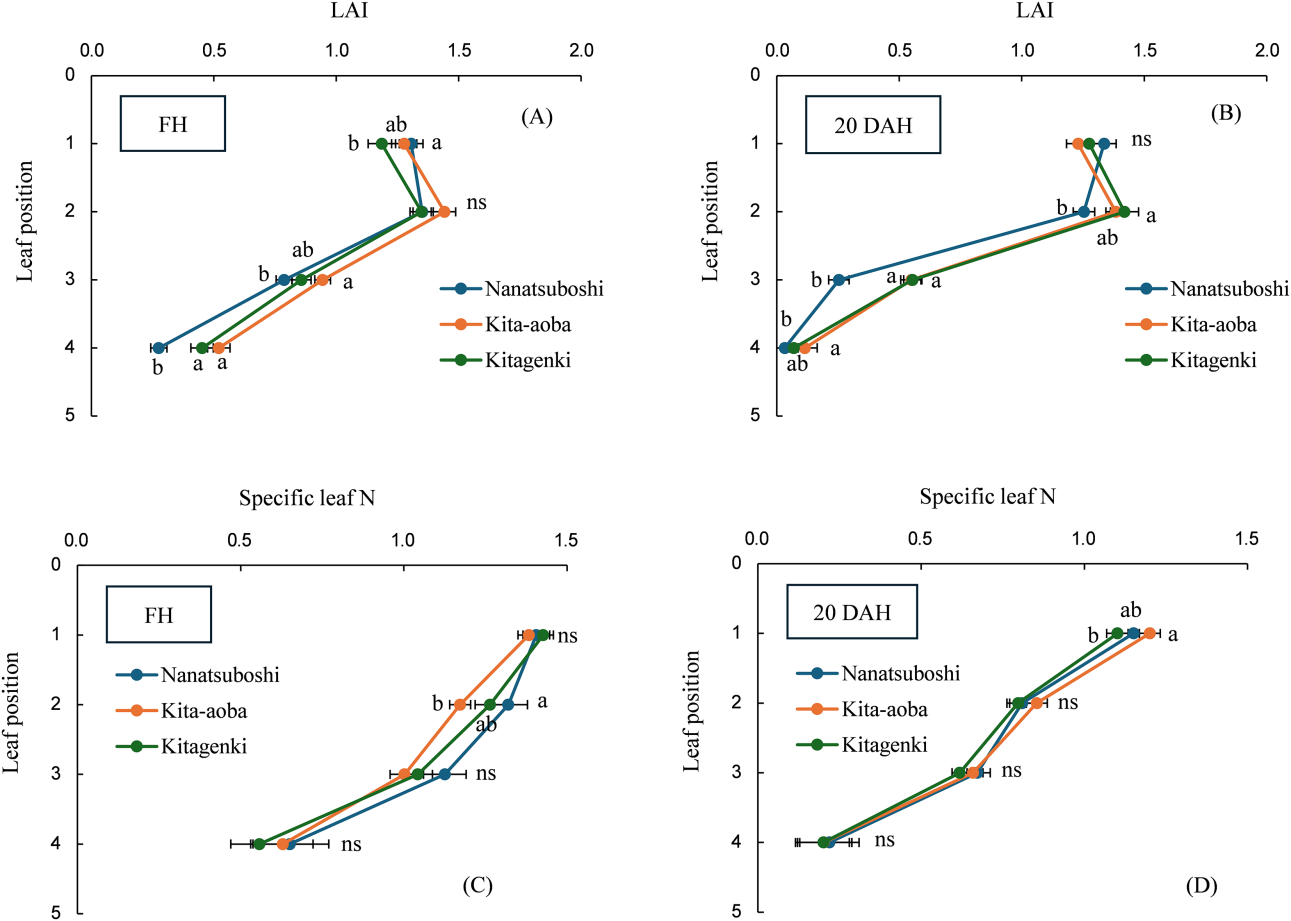
Leaf area index (LAI) measured at FH **(A)** and 20 DAH **(B)** and specific leaf N measured at FH **(C)** and 20 DAH **(D)**. The data are averaged over 5 years (2016–2020). Different lowercase letters among the three varieties at each leaf position indicate statistically significant differences (p < 0.05) based on Tukey’s multiple comparison analysis following two-way analysis of variance (ANOVA); ns, not significant by two-way ANOVA. DAH, days after the full-heading stage; FH, full-heading stage.

The LAI was not significantly different between Kitagenki and Kita-aoba on any leaf position at FH and 20 DAH (**Figures 3A, B**). Specific leaf N was not significantly different between Kitagenki and Kita-aoba at FH (**Figure 3C**), whereas Kitagenki revealed markedly lower specific leaf N in the first leaf than Kita-aoba at 20 DAH (**Figure 3D**). Compared with Nanatsuboshi, Kitagenki exhibited a significantly higher LAI on the fourth leaf at FH and the second and third leaves at 20 DAH, whereas no substantial differences were noted in specific leaf N.

### 3.5 Leaf inclination angle, leaf blade length, and leaf blade width

**Figure 4:**
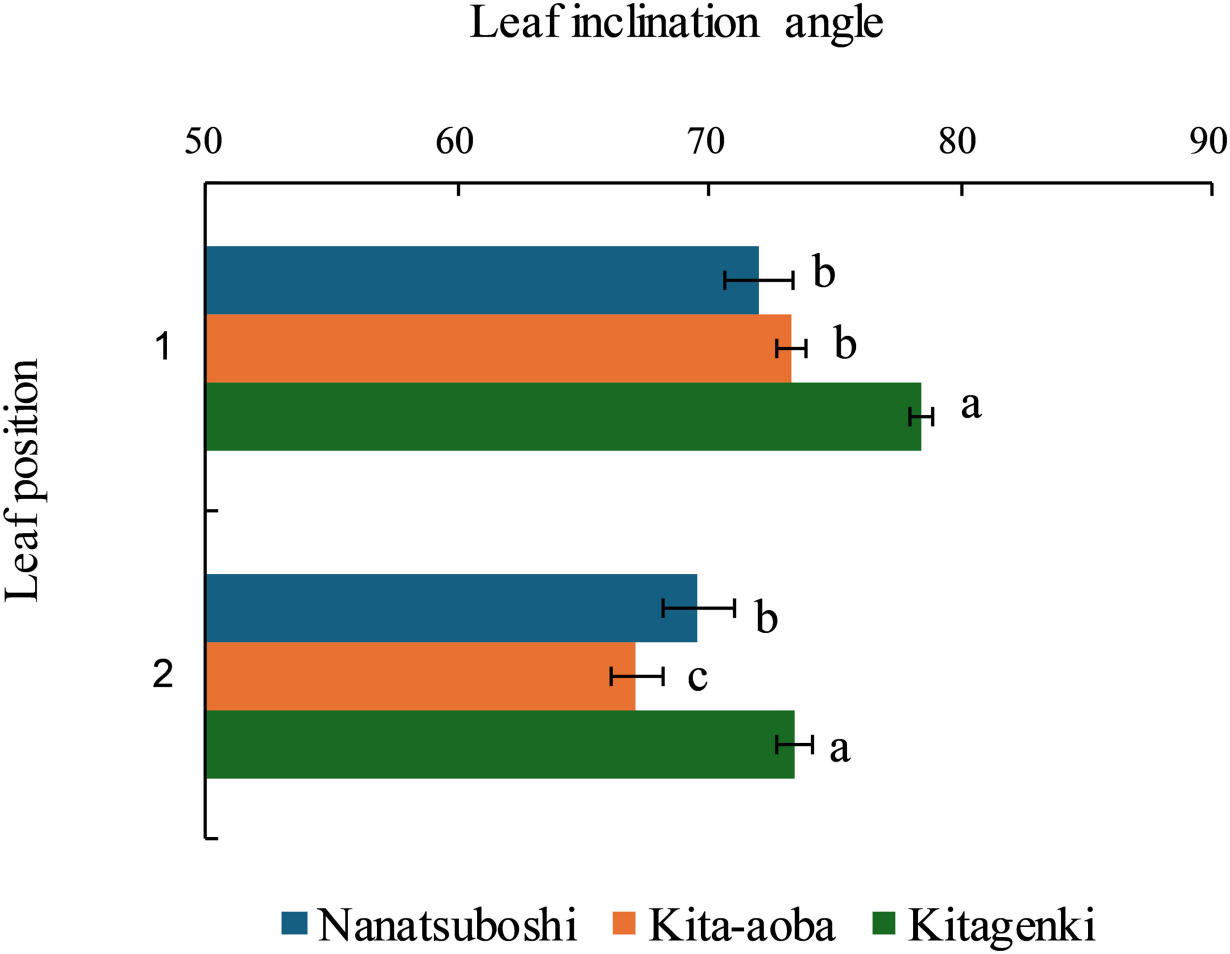
Leaf inclination angles from the horizon of the upper two leaves (1 and 2) of the rice plants were measured during the early grain-filling stages. Data are averaged over 5 years (2016–2020). Horizontal bars indicate the standard error. Different lowercase letters among the three varieties at each leaf position indicate statistically significant differences (p < 0.05) based on Tukey’s multiple comparison analysis following two-way analysis of variance (ANOVA).

The leaf inclination angles of the first and second leaves were the highest in Kitagenki among the three varieties (**Figure 4**). The leaf blade length of Kitagenki was comparable to that of Kita-aoba and markedly higher than that of Nanatsuboshi at all three leaf positions (**Supplementary Table S2**). The upper three leaves of Kitagenki were significantly narrower than those of Kita-aoba.

### 3.6 Relative light interception rate

**Figure 5:**
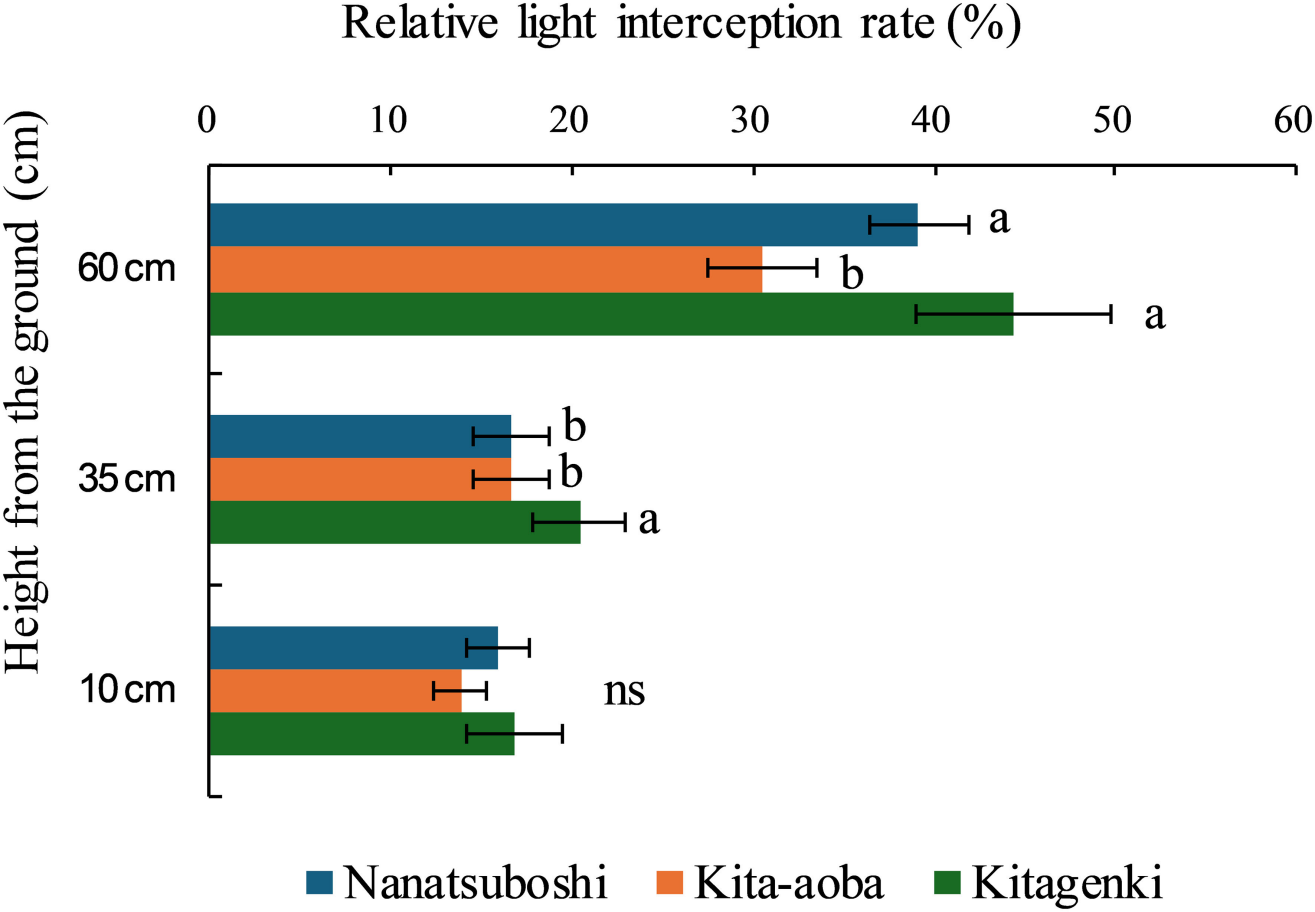
Relative light interception rates among rice plant rows sampled during the early grain-filling stage. The data are averaged over 3 years (2016–2018). Horizontal bars indicate standard errors. Different lowercase letters among the three varieties at each height indicate statistically significant differences (p < 0.05) based on Tukey’s multiple comparison analysis following two-way analysis of variance (ANOVA); ns, not significant by two-way ANOVA.

Relative light interception rate was significantly higher in Kitagenki than in Kita-aoba, by 45.4% at 60 cm and 22.4% at 35 cm, respectively (**Figure 5**).

### 3.7 Leaf photosynthetic rate and stomatal conductance

**Figure 6:**
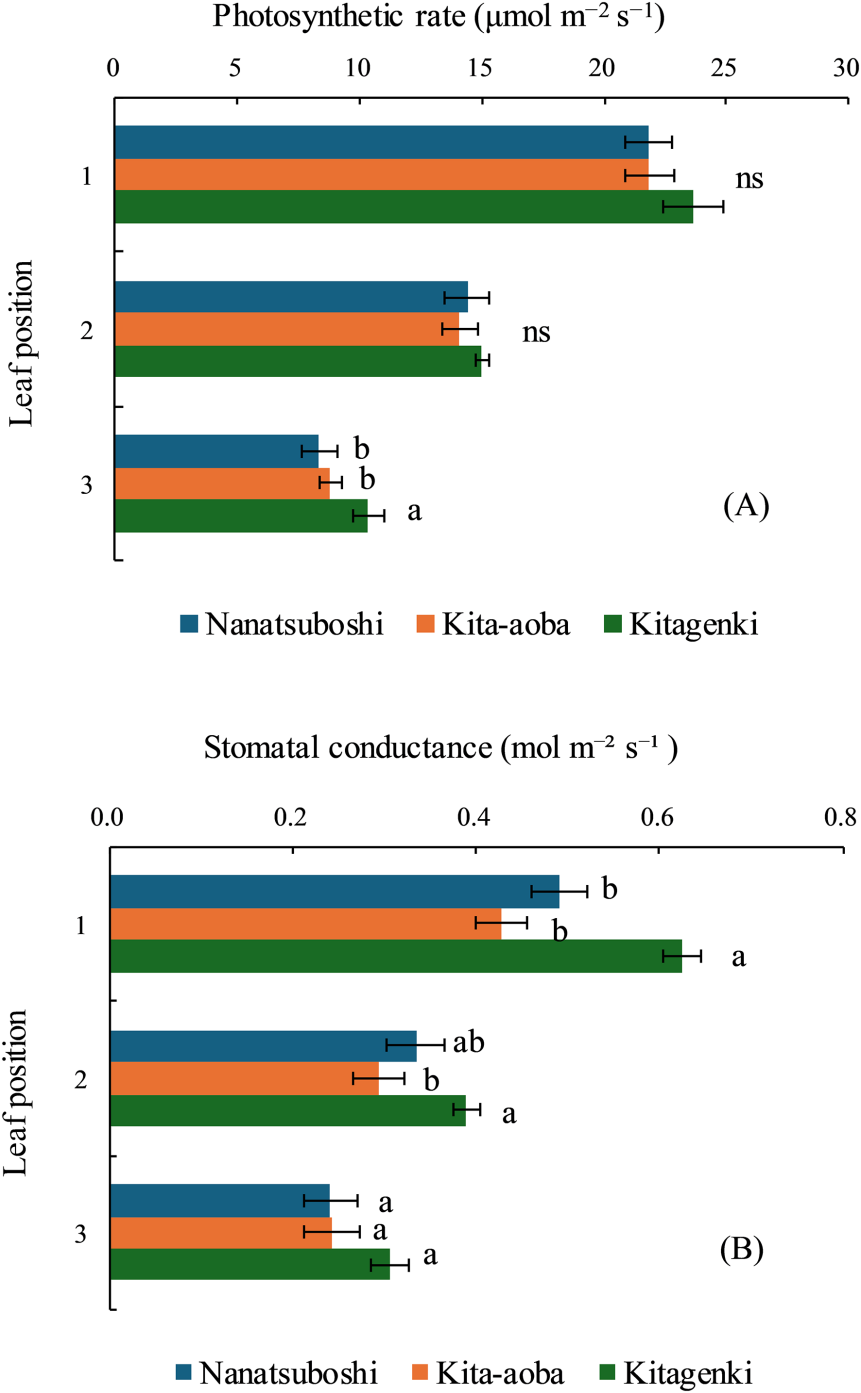
Leaf photosynthetic rate **(A)** and stomatal conductance **(B)** of rice plants during the early grain-filling stage. The data are averaged over 2 years (2017–2018). Horizontal bars indicate standard errors. Different lowercase letters among the three varieties at each leaf position indicate statistically significant differences (p < 0.05) based on Tukey’s multiple comparison analysis following two-way analysis of variance (ANOVA); ns, not significant by two-way ANOVA.

During early grain-filling stages, leaf photosynthetic rate of Kitagenki was markedly higher than that of Kita-aoba by 17.7% on the third leaf and tended to be higher than that of Kita-aoba by 8.3% and 6.5% in the first and second leaves, respectively (**Figure 6A**). Stomatal conductance was markedly higher in Kitagenki than in Kita-aoba by 46.4% and 32.9% in the first and second leaves, respectively, and tended to be higher in Kitagenki by 25.4% in the third leaf (p < 0.10, **Figure 6B**).

### 3.8 ^13^C distribution

**Figure 7:**
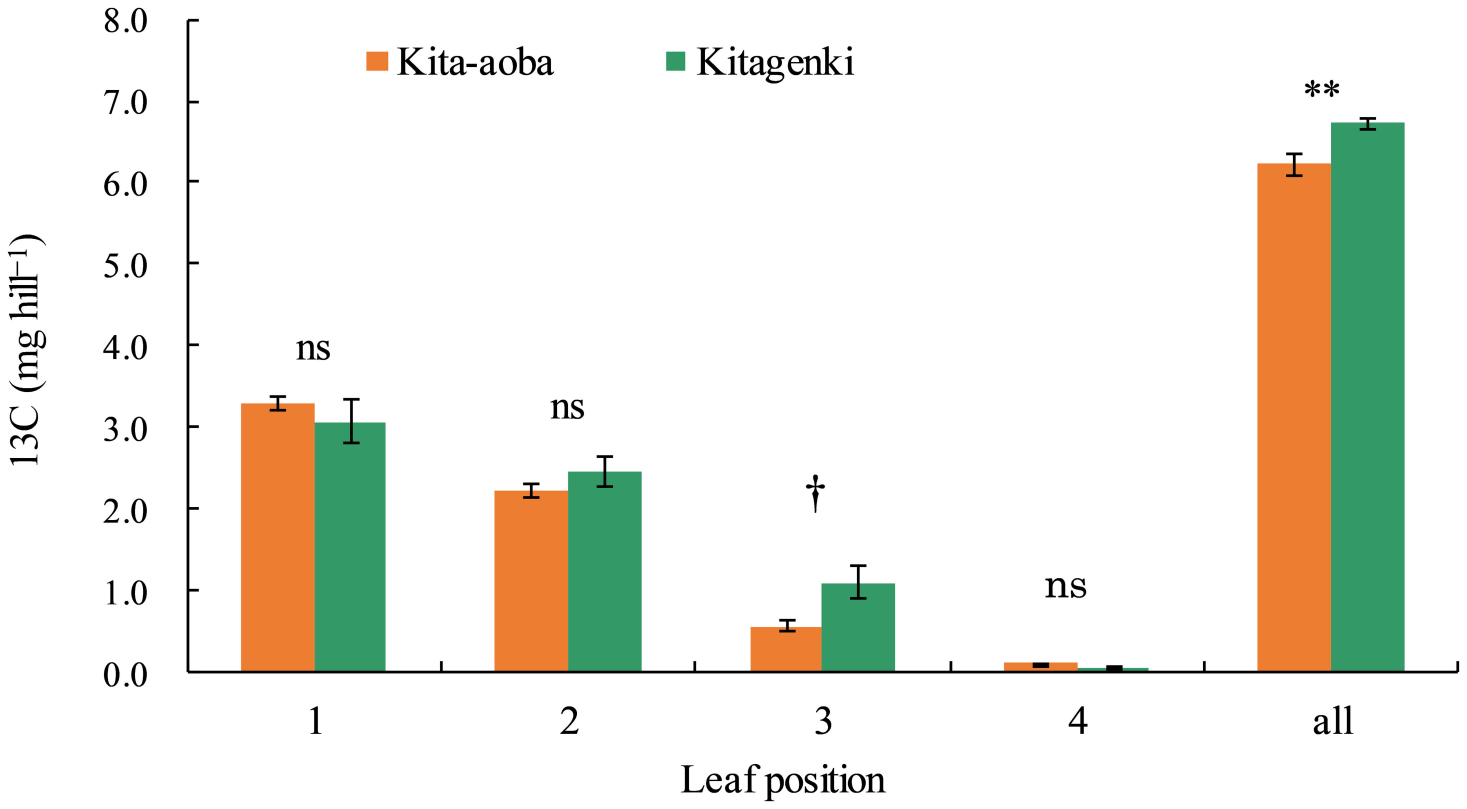
^13^C distribution at each position of the rice leaf blade sampled 1 h after ^13^C feeding in 2020. ^13^C was applied 12 days after the full heading stage (DAH). Vertical bars indicate standard errors. † and ** in each part indicate statistically significant differences at p < 0.10 and p < 0.01, respectively; ns, not significant by one-way analysis of variance (ANOVA).

Kitagenki revealed markedly higher ^13^C distribution by 92% and 8.1% on the third and whole leaves, respectively, at 1 h following feeding than Kita-aoba (**Figure 7**).

**Figure 8:**
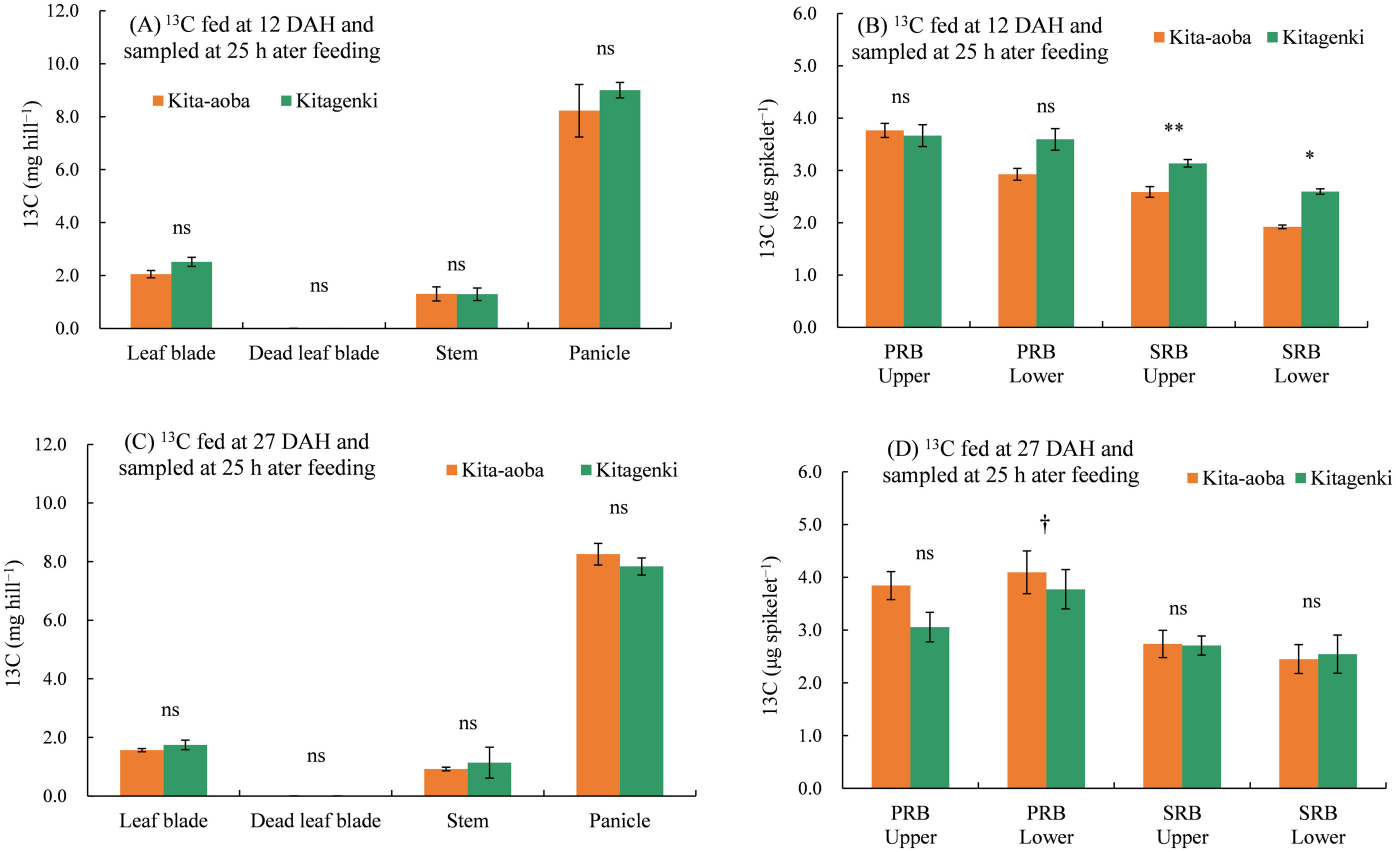
^13^C distribution in each part of rice plants sampled 25 h after feeding in 2020. ^13^C was applied at 12 **(A, B)** and 27 DAH **(C, D)**. Vertical bars indicate standard errors. †, *, and ** in each part at each feeding time indicate significant differences at p < 0.10, p < 0.05, and p < 0.01, respectively; ns, not significant by one-way analysis of variance (ANOVA). DAH, days after the full-heading stage; PRB, primary rachis branch; SRB, secondary rachis branch.

As for the ^13^C distribution in various parts 25 h following feeding, ^13^C fed at 12 and 27 DAH was not markedly different between Kitagenki and Kita-aoba on the leaf blades, dead leaf blades, stems, and panicles (**Figures 8A, C**). However, ^13^C per spikelet fed at 12 DAH was substantially higher in Kitagenki than in Kita-aoba, by 21.2% and 35.1% in the SRB upper and lower parts, respectively, and tended to be higher in Kita-aoba by 22.8% in the PRB lower parts (**Figure 8B**). Contrastingly, ^13^C per spikelet fed at 27 DAH was significantly lower in Kitagenki than in Kita-aoba by 7.9% in the PRB lower parts and tended to be lower in Kitagenki than in Kita-aoba by 22.5% in the PRB upper parts. Contrastingly, it was comparable between Kitagenki and Kita-aoba in the SRB upper and lower parts (**Figure 8D**).

### 3.9 Grain-filling of the spikelet on various positions in the panicle

**Figure 9:**
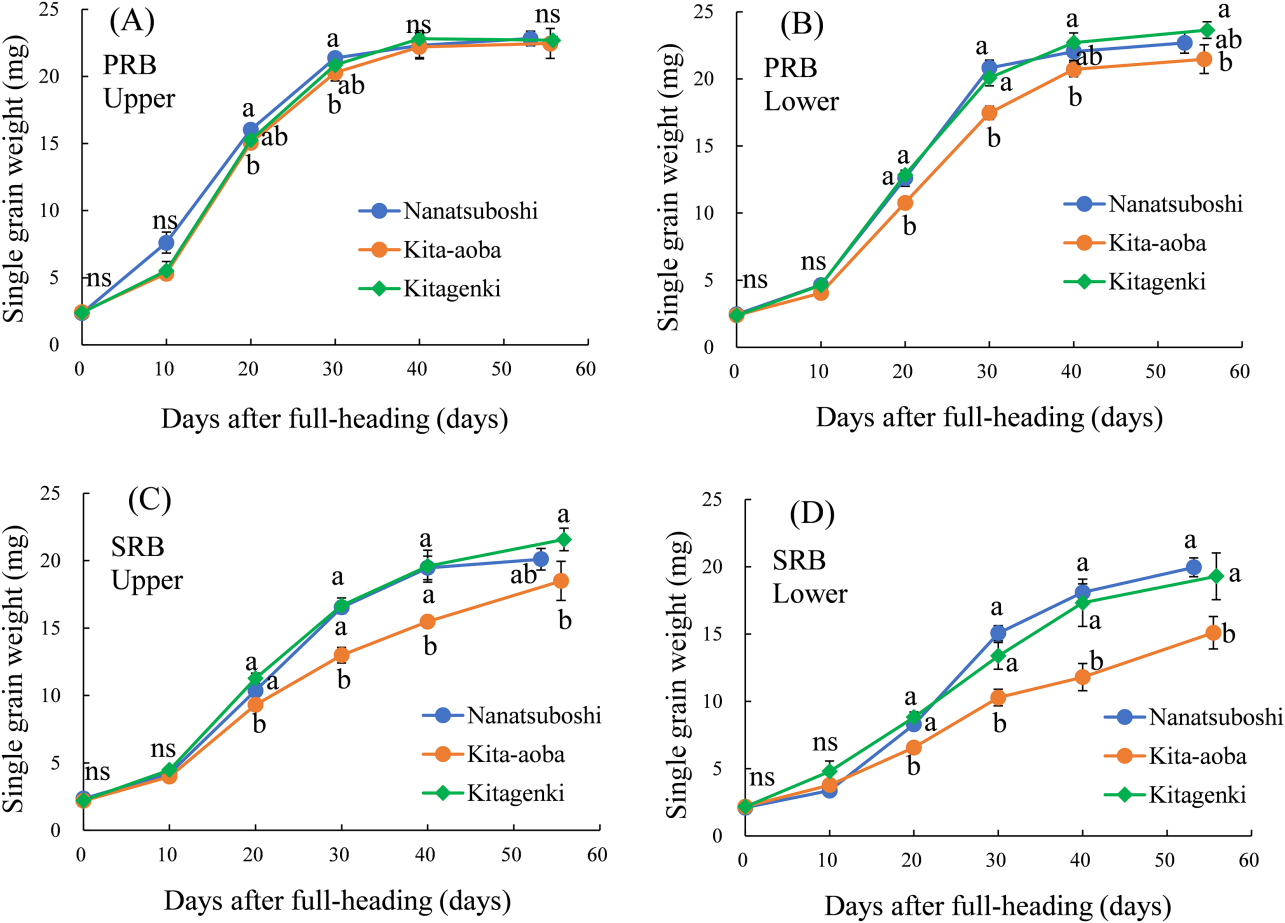
Single grain weight in the PRB upper parts **(A)**, PRB lower parts **(B)**, SRB upper parts **(C)**, and SRB lower parts **(D)** sampled during the grain-filling stage. The data were averaged over 2 years (2016–2017). Vertical bars indicate the standard error. Different lowercase letters among the three varieties at each sampling time indicate statistically significant differences (p < 0.05) based on Tukey’s multiple comparison analysis following two-way analysis of variance (ANOVA); ns, not significant by two-way ANOVA. DAH, days after the full-heading stage; PRB, primary rachis branch; SRB, secondary rachis branch.

During the entire grain-filling stage, the weight of single grain weight in the upper parts of the PRB was not markedly different between Kitagenki and Kita-aoba (**Figure 9A**). In contrast, Kitagenki had markedly heavier single grain weight than Kita-aoba from 20 DAH to M in the upper parts of the PRB, and upper and lower parts of the SRB (**Figures 9B-D**).

At M, filled spikelet number in Kitagenki was markedly lower by 10.7% in the PRB upper parts and significantly higher by 25.9% in the SRB upper parts than that of Kita-aoba (**Supplementary Table S3**). Similarly, filled grain weight of Kitagenki was substantially lower by 8.4% in the PRB upper parts and markedly higher by 31.7% in the SRB upper parts than that of Kita-aoba (**Supplementary Table S3**).

### 3.10 Relationship between traits

**Figure 10:**
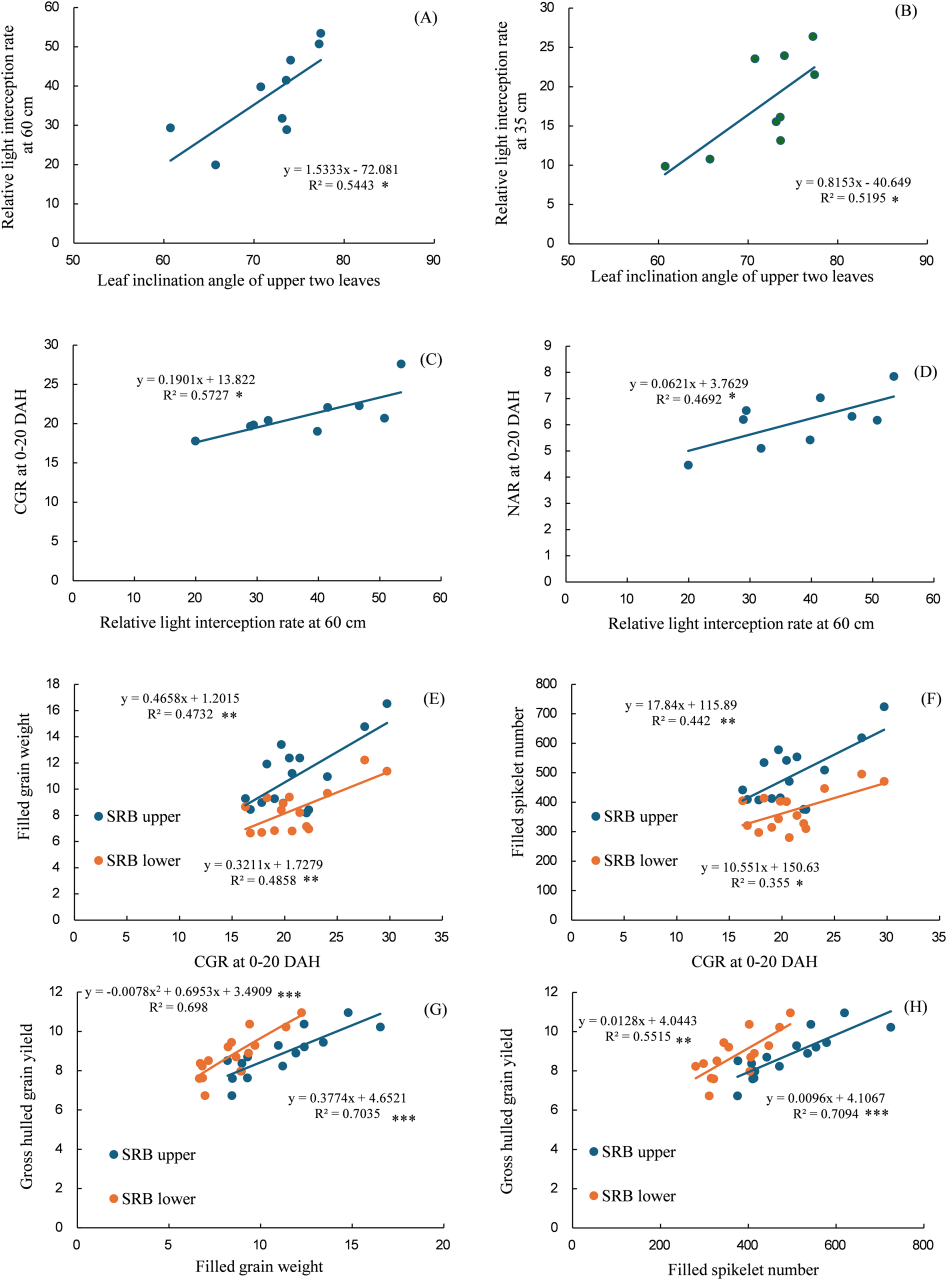
Relationships between the leaf inclination angles of the upper two leaves and the relative light interception rate at 60 cm **(A)**, leaf inclination angle of the upper two leaves and relative light interception rate at 35 cm **(B)**, relative light interception rate at 60 cm and CGR at 0–20 DAH **(C)**, and relative light interception rate at 60 cm and NAR at 0–20 DAH **(D)**, CGR at 0–20 DAH and filled grain weight on SRB upper and lower parts **(E)**, CGR at 0–20 DAH and filled spikelet number on SRB upper and lower parts **(F)**, filled grain weight on SRB upper and lower parts and gross hulled grain yield **(G)**, and filled spikelet number on SRB upper and lower parts and gross hulled grain yield **(H)**. The leaf inclination angle was the average value of the two uppermost leaves. The data of 3 years (2016–2018) were used for **(A-D)**, whereas that of 5 years (2016–2020) were used for **(E-H)**. *, **, and *** indicates significant regression at p < 0.05, p < 0.01, and p < 0.001, respectively. CGR, crop growth rate; DAH, days after the full-heading stage; NAR, net assimilation rate; PRB, primary rachis branch; SRB, secondary rachis branch.

A significant and positive linear relationship was observed between the leaf inclination angle of the upper two leaves and the relative light interception rate at 60 and 35 cm (**Figures 10A, B**), relative light interception rate at 60 cm, and CGR and NAR at 0–20 DAH (**Figures 10C, D**). A significant and positive linear relationship was observed between: CGR at 0–20 DAH and filled grain weight and filled spikelet number in the SRB upper and lower parts (**Figures 10E, F**), filled grain weight and filled spikelet number in the SRB upper and lower parts and gross hulled grain yield (**Figures 10G, H**).

**Figure 11:**
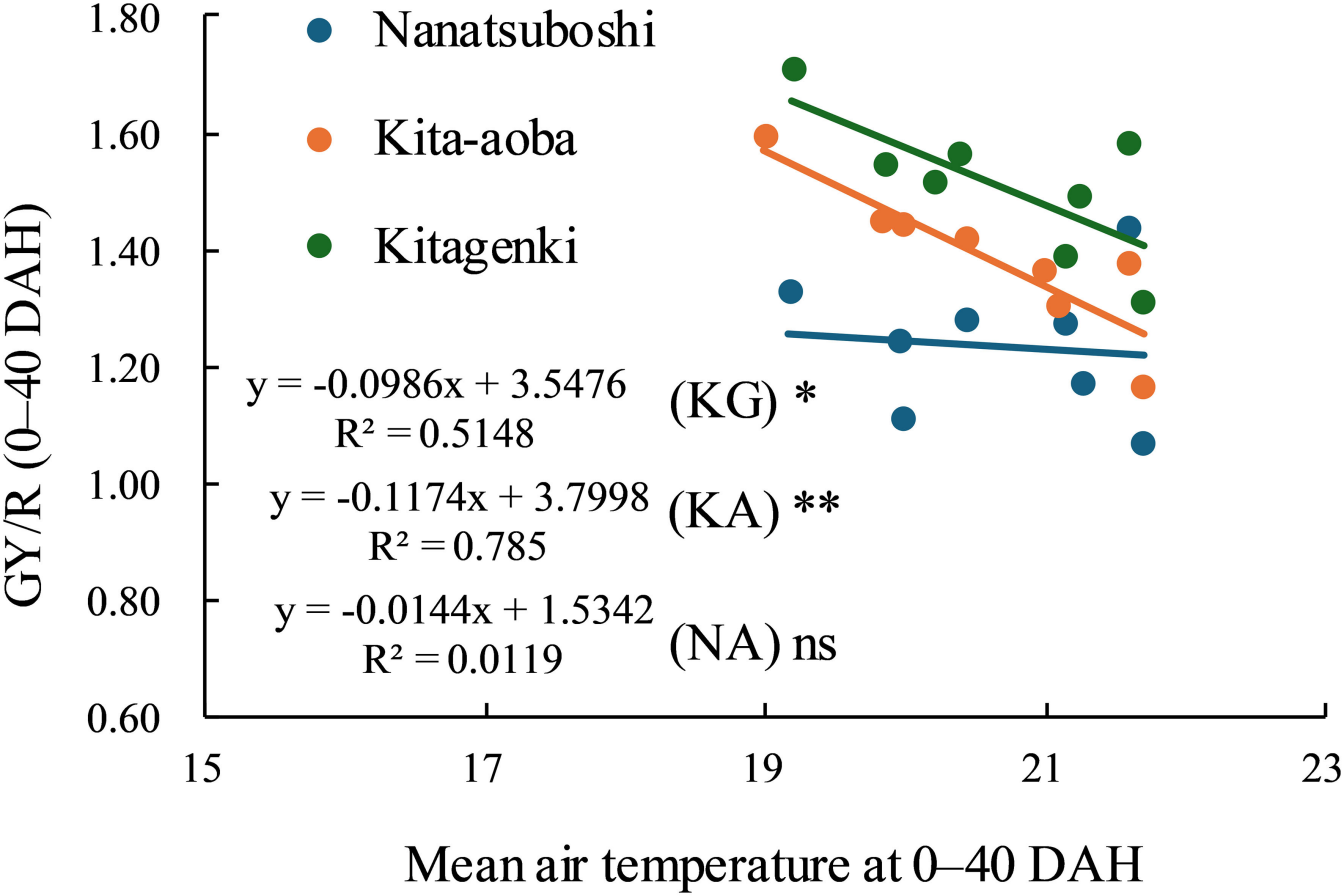
Relationship between mean air temperature during 0–40 DAH and grain yield per cumulative solar radiation at 0–40 DAH. * and ** at each variety indicate significant regression at p < 0.05 and p < 0.01, respectively; ns = not significant regression. DAH, days after the full-heading stage; GY, grain yield; KA, Kita-aoba; KG, Kitagenki; NA, Nanatsuboshi; R, cumulative solar radiation at 0–40 DAH.

A significant negative linear relationship was observed between the mean air temperature at 0–40 DAH and GY/R for Kitagenki and Kita-aoba, and a flat but weak relationship for Nanatsuboshi (**Figure 11**). However, GY/R was greater in Kitagenki than in Kita-aoba and Nanatsuboshi under fluctuated air temperature (approximately within 19–22°C) in the 8-year field experiments. GR/R was the highest in Kitagenki, followed by Kita-aoba, and the lowest in Nanatsuboshi at lower mean air temperature at approximately 19°C, whereas it was higher in Kitagenki than in Kita-aoba and Nanatsuboshi at higher mean air temperature of approximately 22°C.

Additionally, a significant negative linear relationship was noted between the mean air temperature at 0–40 DAH and the filled-grain percentage/R for all three varieties (**Supplementary Figure S4**). However, filled-grain percentage/R was greater in Kitagenki than in Kita-aoba under fluctuated air temperature (approximately within 19–22°C) in the 8-year field experiments.

## 4 Discussion

Our findings support the hypothesis that superior canopy architecture and carbon assimilation of the lower leaves improve biomass productivity in Kitagenki. Regarding canopy morphological and physiological traits, our results showed that the superior canopy architecture of Kitagenki, characterized by more erect and narrower upper leaves (**Figure 4**, **Supplementary Table S2**), facilitated greater light transmission inside the canopy than that of Kita-aoba (**Figures 10A, B**). The shorter culm length in Kitagenki is a superior canopy structure that could mitigate lodging (**Supplementary Table S1**). Therefore, Kitagenki demonstrated greater light availability in the lower parts of the canopy than Kita-aoba (**Figure 5**). This contributed to the increase in NAR and CGR during the early grain-filling stage (**Figures 2A, C**, **10C, D**). Our findings are consistent with those of previous studies on the close relationship between leaf inclination angle and canopy light distribution (Ouyang et al., 2021) and between relative light intensity and NAR (Murata, 1975). NAR is influenced by the canopy architecture and light interception characteristics and the photosynthesis of each leaf in the canopy (San-oh et al., 2004). In addition to superior canopy architecture and higher light interception, Kitagenki demonstrated a higher photosynthetic capacity for the third leaf than Kita-aoba (**Figure 6A**), which contributed to its high NAR (**Figure 2C**). Additionally, the findings of ^13^C assimilation by leaves (**Figure 7**) provided direct evidence that higher carbon assimilation by lower leaves markedly contributed to increased whole-canopy carbon assimilation in Kitagenki. Our result of ^13^C assimilation by leaves is different those reported by Saitoh et al. (2002) who reported that flag leaf contributes more to canopy photosynthesis in indica-type HYV Takanari than standard variety Nipponbare in warmer region in Japan. One possible cause of the discrepancy is lower LAI observed in the present study than those reported by Saitoh et al. (2002), which may have allowed greater light transmission to the lower parts of the canopy. Similar to our results, Cheng et al. (2024) revealed that a high-yielding ideal plant type variety exhibited better light interception within canopy and higher canopy photosynthesis than conventional variety in the subtropical region of China. However, the contribution of carbon assimilation of lower leaves have not been quantitatively evaluated in cold region. Therefore, our results provided the novel insight on the importance of lower leaves for canopy photosynthesis in cold region.

Gu et al. (2017) reported that canopy light and N distribution were closely associated with canopy photosynthesis. In the current study, Kitagenki and Kita-aoba exhibited no considerable variations in specific leaf N at FH (**Figure 3C**), whereas light distribution was better in Kitagenki than in Kita-aoba in the lower canopy layers (**Figure 5**). Thus, better light distribution inside canopy and not leaf N distribution is responsible for the high canopy photosynthesis in Kitagenki during the early grain-filing stages. Leaf photosynthetic potential is mostly explained by stomatal conductance and leaf N content (Takai et al., 2010). At the single-leaf level, higher stomatal conductance (**Figure 6B**), not leaf N content (**Figure 3C**) may contribute to the high carbon assimilation (**Figure 7**) and photosynthetic rate of Kitagenki (**Figure 6A**). Consequently, Kitagenki showed stably high biomass productivity during the early grain-filling stages by improving the NAR while maintaining an LAI comparable to that of Kita-aoba (**Figures 2A–C**, **Supplementary Figure S5**). Our results were generally in accordance with that of Anzoua et al. (2010), who used rice varieties released during 1905–1988. They stated that modern rice varieties in the Hokkaido region have improved canopy structure and light capture while having a high LAI compared with older varieties, leading to yield improvement. However, our study used more recent HYV with higher yield potentials (gross hulled grain yield of 12 t ha^−1^ in Yagioka et al., 2022 versus 7.5 t ha^−1^ in Anzoua et al., 2010). The yield potential (rough grain yield of 14.5 t ha^−1^ in Yagioka et al., 2022) were also higher than that of Longdao-18 achieved in the northernmost limit of rice cultivation in Heilongjiang province in China (13.4 t ha^−1^; Shahbaz Farooq et al., 2021). Therefore, our study emphasizes the criticality of superior canopy architecture and light interception with a high LAI for breeding future HYV to break the yield ceiling in cold region.

An adequate carbohydrate supply during the early grain-filing stages in Kitagenki (**Figure 2A**) led to a high grain-filing ability (**Supplementary Figure S3B**) and a stably high yield (**Figure 1**) compared to Kita-aoba. The ^13^C distribution results at 25 h after feeding (**Figure 8B**) indicated that assimilated carbon during the early grain-filling stages (12 DAH) was more preferentially distributed into spikelets on SRB in Kitagenki than in Kita-aoba, which helped in faster panicle growth (**Figure 2D**) and grain-filling of the inferior spikelet from the early grain-filling to maturity stages (**Figure 9**, **Supplementary Table S3**). The results of regression analysis (**Figures 10E–H**) also supported this conclusion. Our results were consistent with those of Shahbaz Farooq et al. (2021) who reported the importance of grain-filling in inferior spikelet for achieving high yield in cold environment in Heilongjiang province in China. In contrast, the less assimilated carbon was distributed into superior spikelets (PRB) in Kitagenki as compared with Kita-aoba (**Figure 8D**, **Supplementary Table S3**). This suggests that superior spikelets has completed the grain-filling at the earlier growth stages in Kitagenki compared to Kita-aoba (**Figure 9**), which were primarily caused by adequate carbohydrate supply during the early grain-filling stages (**Figure 2A**, **Supplementary Figure S5**).

The current study confirmed a consistently higher Kitagenki yield than HYV: Kita-aoba and SYV: Nanatsuboshi in an 8-year field experiment (**Figure 1**, **Supplementary Table S1**), which is consistent with our previous findings from the first 5 years of field experiments (2014–2018; Yagioka et al., 2021). The yield level in the present study is lower than our previous study (Yagioka et al., 2022), due to the lower N application rate in the present study (105 kgN ha^−1^; 105–225 kgN ha^−1^ in Yagioka et al., 2022). This indicates the potential of further increasing yield potential of Kitagenki by optimizing crop management practices. The maximum grain yield of Kitagenki in the present study (13.1 t ha^−1^, **Supplementary Table S1**) is comparable with that of Longdao-18 cultivated in Heilongjiang province in China (13.4 t ha^−1^; Shahbaz Farooq et al., 2021). However, grain yield was stably higher in Kitagenki (9.8–13.1 t ha^−1^, **Supplementary Table S1**) than Longdao-18 (7.4–13.4 t ha^−1^; Shahbaz Farooq et al., 2021). In the present study, adding an additional 3 years of field data enabled further analysis of the grain yield response to different climatic conditions. The analysis results (**Figure 11**) suggest that Kitagenki has superior yield performance and adaptability to fluctuating air temperature during the grain-filing stages compared to Kita-aoba and Nanatsuboshi. Similar varietal variations between Kitagenki and Kita-aoba were noted in the relationship between filled-grain percentage/R and mean air temperature (**Supplementary Figure S4**), suggesting that the higher grain-filling ability of Kitagenki contributes to a higher yield stability than that of Kita-aoba. As already discussed, stably superior biomass productivity of Kitagenki during the early grain-filing stages (**Supplementary Figure S5A**), also contributes to stabilize the grain yield by enhancing grain-filling ability in inferior spikelet (**Figures 10E–H**). As a results, Kitagenki showed higher yield performance under higher air temperature during grain-filing stages than Kita-aoba (**Figure 11**). Although this study did not elucidate the detailed mechanisms of the adaptability, the higher stomatal conductance of Kitagenki (**Figure 6B**) may possibly contribute to adaptation to higher air temperatures by lowering the canopy temperature (Takai et al., 2010).

Developing HYV with a large sink capacity is the most important trait to achieve a high grain yield in the Hokkaido region as breeding strategies for future HYV, whereas improving grain-filling ability by source improvement is required to satisfy the enlarged sink capacity (Hayashi et al., 2012; Yagioka et al., 2021). Between two source components, ΔW during grain-filling stages is quantitatively more important than NSC at FH in the Hokkaido region (Kusutani, 1988; Yagioka et al., 2021) because NSC at FH was smaller in colder than in warmer regions. The current study further showed that high source ability during the early grain-filling stage, supported by superior canopy architecture (e.g., more erect and narrower upper leaves and shorter culm length), higher light interception, and carbon assimilation of lower leaves, contributes to the high grain-filling ability and stable high yield. Furthermore, the high yield performance under fluctuating air temperatures during the grain-filling stages is beneficial because increasing the air temperature negatively influences rice grain yield (Song et al., 2022). These superior traits of the recent HYV could be used as indicators for breeding future HYV that can break the yield ceiling in cold regions.

Our study had certain limitations. First, the sink strength was not assessed. Given that sink strength is one of the limiting factors for poor grain filling in HYV (Okamura et al., 2021) and super rice (Fu et al., 2011), low sink strength might be another cause of the lower grain-filling ability of Kita-aoba. Slower C translocation to the inferior spikelet (**Figures 8**, **9**) suggests this possibility. Second, it is unknown on the GY/R response of Kitagenki to mean air temperature outside our experimental range (<19°C and/or >22°C). Third, the detailed mechanisms of high radiation use efficiency (e.g., GY/R) and/or higher adaptability to fluctuating air temperatures in Kitagenki should be elucidated. Further emphasis should be placed on the adaptability of future HYV to higher air temperatures, considering that the global surface temperature is predicted to continue to increase until at least the mid-century (IPCC, 2021). Cross-locational analysis that compares HYV in the Hokkaido region and warmer regions, such as the Honsyu region, the main island of Japan, can further provide the key traits for developing future HYV that have high yield stability under increasing air temperatures.

## 5 Conclusion

The grain yield of Kitagenki was consistently higher than that of Kita-aoba, owing to its higher percentage of filled grains. Kitagenki had higher biomass productivity than Kita-aoba during the early grain-filling stages (0–20 DAH) due to a higher NAR while maintaining LAI. The superior canopy architecture (e.g., more erect and narrower upper leaves and shorter culm length), higher light interception inside the canopy, and higher photosynthetic performance and carbon assimilation of the lower leaves of Kitagenki contributed to its higher canopy productivity compared with Kita-aoba. The elevated carbohydrate supply during the early grain-filling stages in Kitagenki facilitated its faster translocation into the inferior spikelet, resulting in a higher grain-filling ability than that in Kita-aoba. This further contributed to the consistently higher GY/R of Kitagenki under fluctuating air temperatures during the grain-filling stages compared with Kita-aoba. These superior canopy morphological and physiological traits could be used as indicators for breeding future HYV that could break the yield ceiling in cold regions.

## Data Availability

The raw data supporting the conclusions of this article will be made available by the authors, without undue reservation.
