# Supplementary material for: Elucidation of morphological and physiological traits contributing to high biomass productivity and consistently high yield in the high-yielding rice variety Kitagenki
Source: Front Plant Sci. 2025 Nov 19;16:1710830. doi: 10.3389/fpls.2025.1710830 (PMC12672330; doi:10.3389/fpls.2025.1710830)
Supplement: Supplementary file 1 [file DataSheet1.docx]

Supplementary Material

# Supplementary Figures and Tables

## Supplementary Figures


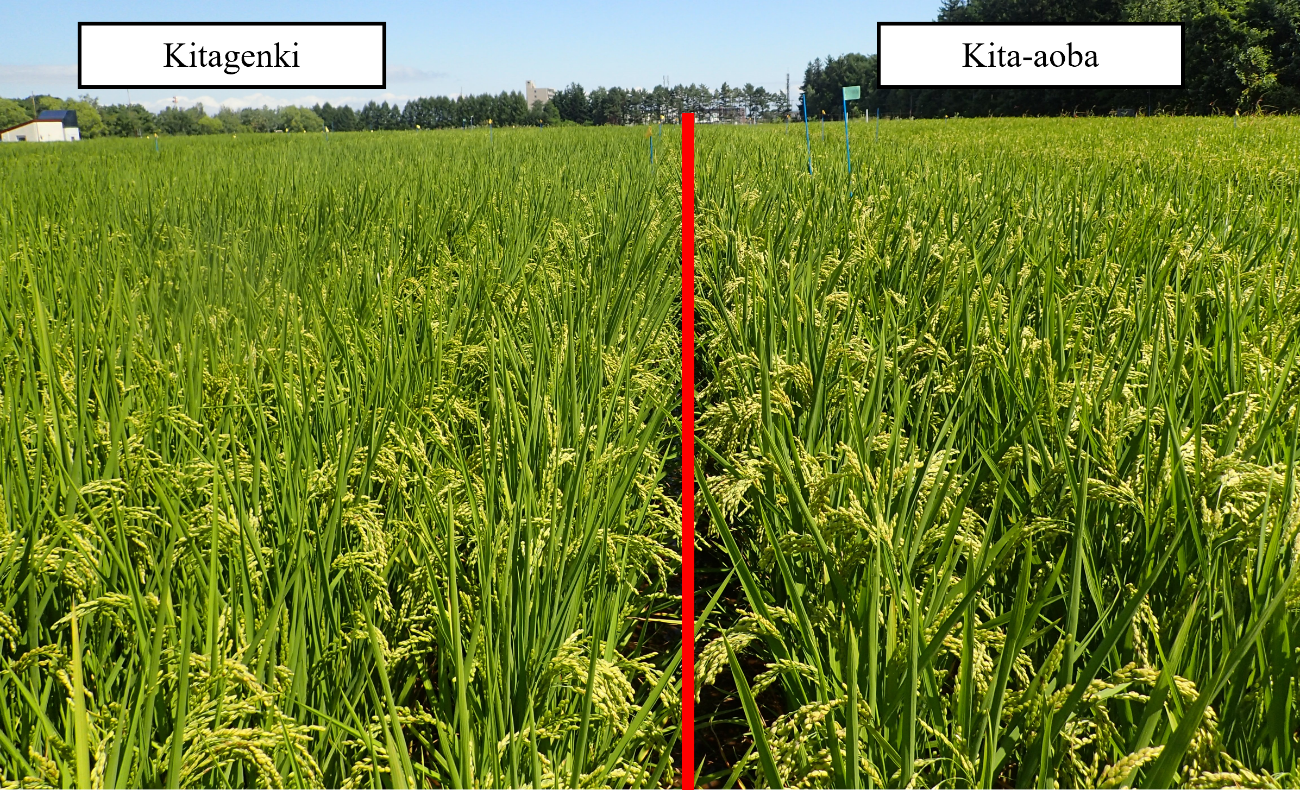


**Supplementary Figure 1.** Images of the canopy architectures of Kitagenki (left) and Kita-aoba (right) during the early grain-filling stage in 2020.


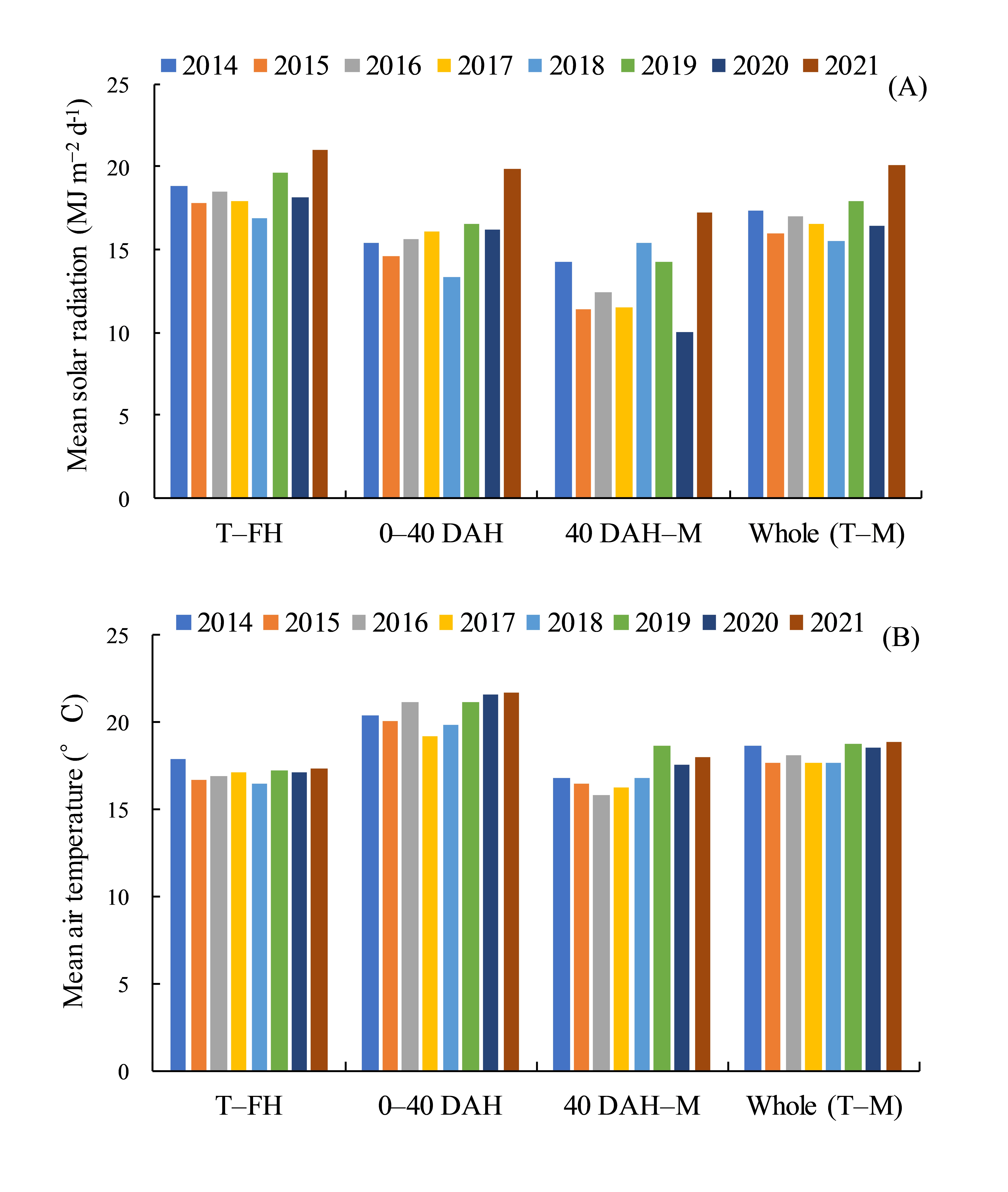


**Supplementary Figure 2.** Mean solar radiation (A) and mean air temperature (B) at T–FH, 0–40 DAH, 40 DAH–M, and whole (T–M). Data are averaged across the three varieties. DAH, days after the full-heading stage; FH, full-heading stage; M, maturity stage; T, transplantation stage.


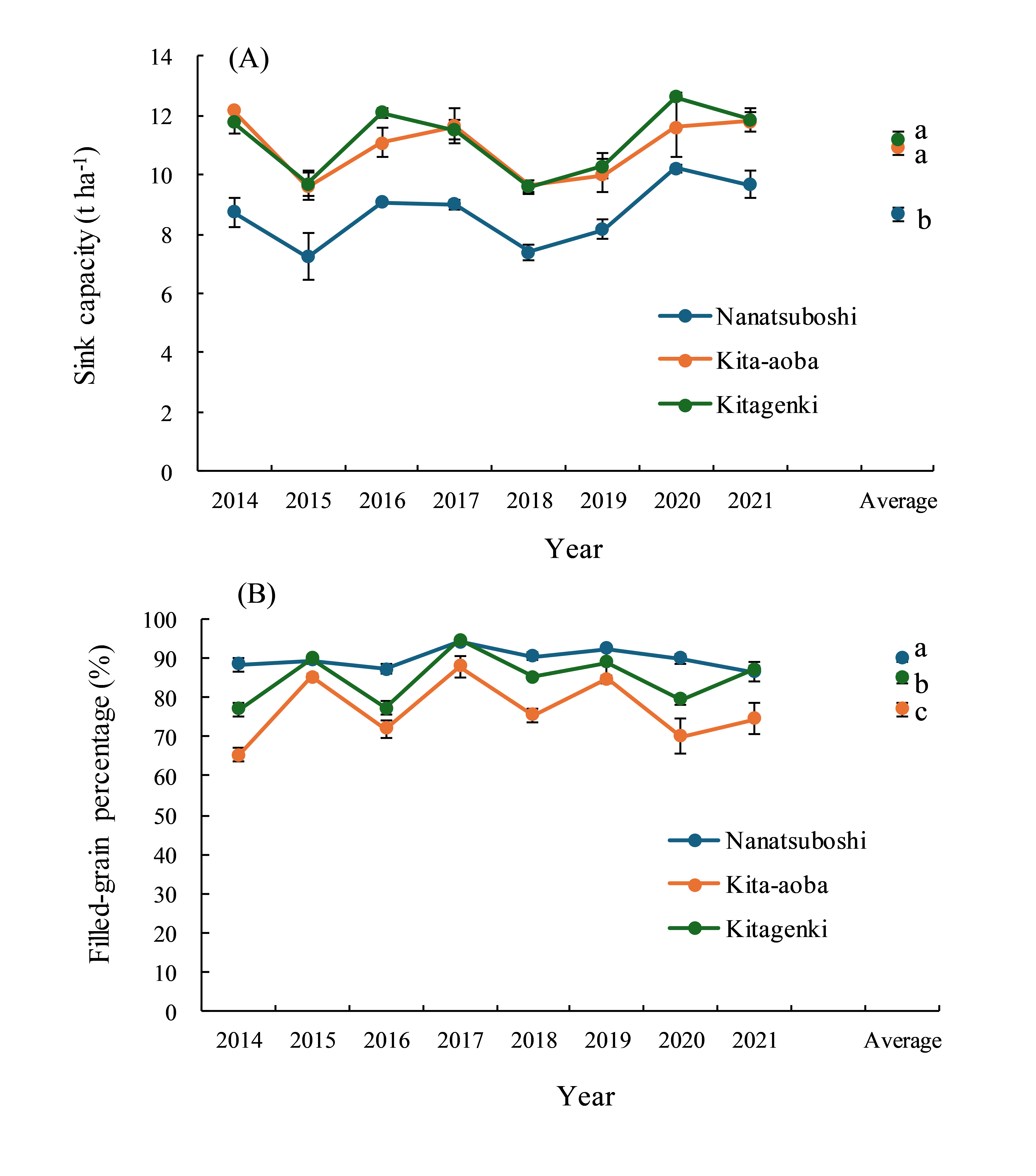


**Supplementary Figure 3.** Variations in sink capacity (A) and filled-grain percentage (B) over 8 years (2014–2021). Vertical bars indicate standard errors. Different lowercase letters among the three varieties for the average value across 8 years indicate statistically significant differences (p < 0.05) based on Tukey’s multiple comparison analysis following two-way analysis of variance (ANOVA). The original sink capacity data for 2014–2018 are obtained from Yagioka et al. (2021), with a modification to reflect the grain moisture content (15% in the current study).


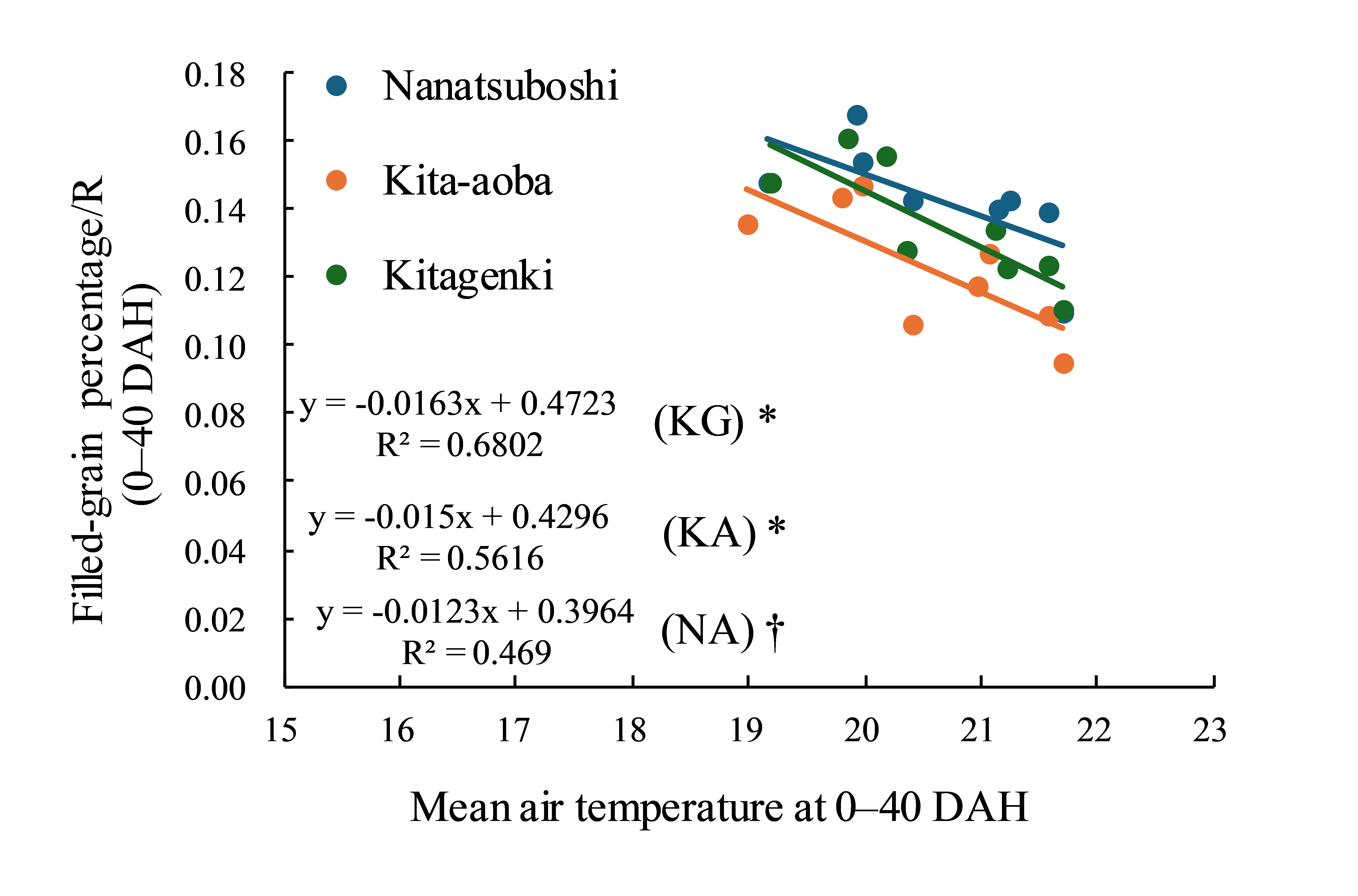


**Supplementary Figure 4.** Relationship between mean air temperature at 0–40 DAH and filled-grain percentage per cumulative solar radiation at 0–40 DAH. † and * at each variety indicate statistically significant regression at p < 0.10 and p < 0.05, respectively. DAH, days after the full-heading stage; KA, Kita-aoba; KG, Kitagenki; NA, Nanatsuboshi; R, cumulative solar radiation at 0–40 DAH.





**Supplementary Figure 5.** Crop growth rate (CGR) (A), mean leaf area index (Mean LAI) (B), and net assimilation rate (NAR) (C) during 2016–2020. Vertical bars indicate the standard error. DAH, days after full-heading stage.

## Supplementary Tables

**Supplementary Table 1.** Growth stage, days from T to FH, grain-filling duration, lodging score, culm length, and rough grain yield.

| Year | Variety | Full-heading day | Harvest day | Days from T to FH | Grain-filling duration | Lodging score (0–5) | | Culm length (cm) | | Rough grain yield (t ha^−1^) | |
| --- | --- | --- | --- | --- | --- | --- | --- | --- | --- | --- | --- |
| 2014 | Nanatsuboshi | 7/28 | 9/16 | 69 | 50 | - |  | - |  | 9.7 |  |
|  | Kita-aoba | 7/28 | 9/16 | 69 | 50 | - |  | - |  | 11.3 |  |
|  | Kitagenki | 7/30 | 9/22 | 71 | 54 | - |  | - |  | 11.9 |  |
| 2015 | Nanatsuboshi | 7/31 | 9/18 | 73 | 49 | 0.1 |  | 78 |  | 7.7 |  |
|  | Kita-aoba | 7/31 | 9/30 | 73 | 61 | 0.4 |  | 81 |  | 10.1 |  |
|  | Kitagenki | 7/30 | 9/30 | 72 | 62 | 0.0 |  | 71 |  | 10.6 |  |
| 2016 | Nanatsuboshi | 8/5 | 9/26 | 78 | 52 | 2.8 |  | 85 |  | 9.7 |  |
|  | Kita-aoba | 8/6 | 9/28 | 79 | 53 | 1.2 |  | 85 |  | 10.9 |  |
|  | Kitagenki | 8/4 | 9/26 | 77 | 53 | 0.0 |  | 78 |  | 12.0 |  |
| 2017 | Nanatsuboshi | 7/30 | 9/22 | 73 | 54 | 0.1 |  | 83 |  | 10.1 |  |
|  | Kita-aoba | 8/2 | 9/29 | 76 | 58 | 1.3 |  | 89 |  | 12.6 |  |
|  | Kitagenki | 7/29 | 9/26 | 72 | 59 | 0.0 |  | 75 |  | 13.1 |  |
| 2018 | Nanatsuboshi | 7/30 | 9/21 | 70 | 53 | 2.7 |  | 73 |  | 8.0 |  |
|  | Kita-aoba | 7/31 | 9/26 | 71 | 57 | 3.3 |  | 78 |  | 9.4 |  |
|  | Kitagenki | 7/30 | 9/26 | 70 | 58 | 0.5 |  | 67 |  | 9.8 |  |
| 2019 | Nanatsuboshi | 7/25 | 9/17 | 64 | 54 | 0.0 |  | 71 |  | 9.2 |  |
|  | Kita-aoba | 7/27 | 9/24 | 66 | 59 | 0.1 |  | 77 |  | 10.8 |  |
|  | Kitagenki | 7/27 | 9/20 | 66 | 55 | 0.0 |  | 72 |  | 11.3 |  |
| 2020 | Nanatsuboshi | 7/31 | 9/23 | 72 | 54 | 1.3 |  | 82 |  | 11.0 |  |
|  | Kita-aoba | 7/31 | 9/27 | 72 | 58 | 1.5 |  | 83 |  | 11.1 |  |
|  | Kitagenki | 7/31 | 9/29 | 72 | 60 | 0.0 |  | 78 |  | 12.4 |  |
| 2021 | Nanatsuboshi | 7/22 | 9/14 | 63 | 54 | 3.7 |  | 81 |  | 10.2 |  |
|  | Kita-aoba | 7/22 | 9/14 | 63 | 54 | 4.3 |  | 82 |  | 11.5 |  |
|  | Kitagenki | 7/22 | 9/21 | 63 | 61 | 0.6 |  | 75 |  | 12.7 |  |
| Average | Nanatsuboshi |  |  | 70 | 53 | 1.5 | a | 79 | b | 9.4 | c |
|  | Kita-aoba |  |  | 71 | 56 | 1.7 | a | 82 | a | 11.0 | b |
|  | Kitagenki |  |  | 70 | 58 | 0.2 | b | 74 | c | 11.7 | a |

^x^ Different lowercase letters among the three varieties indicate statistically significant differences (p < 0.05) based on Tukey’s multiple comparison analysis following two-way analysis of variance (ANOVA). FH, full-heading stage; T, transplantation stage. The original grain yield data from 2014 to 2018 were obtained from Yagioka et al. (2021) with modification of the grain moisture content (15% in the current study).

**Supplementary Table 2.** Leaf blade lengths and widths of the upper three leaves measured at the grain-filling stage. The data are averaged over 3 years (2016–2018).

| Traits | Variety | Leaf positions | | | | | |
| --- | --- | --- | --- | --- | --- | --- | --- |
|  |  | 1 |  | 2 |  | 3 |  |
| Leaf blade length (cm) | Nanatsuboshi | 29.5 | b^x^ | 35.2 | b | 32.4 | b |
|  | Kita-aoba | 34.0 | a | 40.1 | a | 34.2 | ab |
|  | Kitagenki | 35.4 | a | 41.6 | a | 35.6 | a |
| Leaf blade width (cm) | Nanatsuboshi | 1.46 | c | 1.29 | b | 1.09 | b |
|  | Kita-aoba | 1.66 | a | 1.39 | a | 1.19 | a |
|  | Kitagenki | 1.51 | b | 1.20 | c | 1.03 | c |

^x^ Different lowercase letters among the three varieties at each leaf position indicate statistically significant differences (p < 0.05) based on Tukey’s multiple comparison analysis following two-way analysis of variance (ANOVA).

**Supplementary Table 3.** Filled spikelet number and filled grain weight of rice plants sampled at maturity.

| Traits | Variety | PRB | | | |  | SRB | | | |
| --- | --- | --- | --- | --- | --- | --- | --- | --- | --- | --- |
|  |  | Upper | | Lower | |  | Upper | | Lower | |
| Filled spikelet number (hill^−1^) | Nanatsuboshi | 452 | a ^x^ | 357 | a |  | 417 | b | 362 | a |
|  | Kita-aoba | 446 | a | 367 | a |  | 468 | b | 367 | a |
|  | Kitagenki | 398 | b | 364 | a |  | 589 | a | 389 | a |
| Filled grain weight (g hill^−1^) | Nanatsuboshi | 10.3 | a | 8.4 | a |  | 9.0 | c | 7.9 | b |
|  | Kita-aoba | 10.5 | a | 8.5 | a |  | 10.4 | b | 8.2 | ab |
|  | Kitagenki | 9.7 | b | 9.1 | a |  | 13.7 | a | 9.4 | a |

The data are averaged over 5 years (2016–2020). ^x^ Different lowercase letters among the three varieties within each part of the panicle indicate statistically significant differences (p < 0.05) based on Tukey’s multiple comparison analysis, following two-way analysis of variance (ANOVA). PRB, primary rachis branch; SRB, secondary rachis branch.
